# MyD88 polymerization and association to cellular membranes in a yeast heterologous model

**DOI:** 10.1007/s00018-025-05827-1

**Published:** 2025-07-25

**Authors:** Elba del Val, Alejandro Fernández-Vega, María Molina, Víctor J. Cid

**Affiliations:** Department of Microbiology and Parasitology, School of Pharmacy, Complutense University of Madrid, Pza. Ramón y Cajal s/n, Madrid, 28040 Spain

**Keywords:** MyD88, Myddosome, TLR signaling, Humanized yeast, *Saccharomyces cerevisiae*, Innate immunity

## Abstract

MyD88 is a key mediator of Toll-like receptor (TLR) signaling, orchestrating the innate immune response upon stimulation by pathogen-associated molecular patterns (PAMPs). Structurally, MyD88 consists of a Death domain (DD), a 20-amino acid N-terminal extension, and an intermediate (INT) region that connects it to a Toll/Interleukin-1 receptor (TIR) domain. At the core of the signaling complex known as myddosome, MyD88 undergoes homopolymeric interactions to propagate the signal. In this study, we use *Saccharomyces cerevisiae* as a heterologous model to assess the contribution of individual MyD88 domains to self-interaction and subcellular localization. In yeast, MyD88 localizes to endoplasmic reticulum-mitochondria encounter sites (ERMES). Here, we show that its DD is sufficient for attachment to the ERMES. Deletion of its 20 N-terminal residues increased MyD88 stability, shifting its aggregation pattern from patches to filaments. In contrast, a chimeric MyD88 variant bearing the plasma membrane-binding N-terminal extension of TIRAP, another TLR4-associated myddosome component, exhibited diffuse mitochondrial distribution. Moreover, we found that the ERMES-associated dynamin-like protein Dnm1, involved in mitochondrial fission, played a crucial role in MyD88 expression in yeast. On the other hand, the MyD88 TIR domain alone accumulated at lipid droplets in yeast, and its overexpression led to growth impairment and mitochondrial condensation. These findings suggest that MyD88 association with cellular membranes promotes self-assembly, a process essential for functional TLR signaling. Additionally, we adapted a tripartite GFP system to titrate MyD88 homopolymerization in yeast. Using this system, we observed that the oncogenic L252P mutation significantly reduced MyD88 ability to self-interact.

**Supplementary Information:**

The online version contains supplementary material available at 10.1007/s00018-025-05827-1.

## Introduction

Innate immunity and inflammation are triggered upon exposure of cells to pathogen- and damage-associated molecular patterns (PAMPs and DAMPs, respectively) that are detected by PAMP-recognition receptors (PRRs). Among these, Toll-like receptors (TLRs) represent a paradigmatic example. Human tissues express 10 different TLRs that respond to specific threatening molecules, such as bacterial lipopolysaccharide (LPS), detected by TLR4; lipoteichoic acid or lipoproteins, sensed by TLR1, TLR2, and TLR6; bacterial flagellin, recognized by TLR5; and viral nucleic acids, typically detected by TLR3, TLR7, TLR8, and TLR9 [1, 2]. The stimulation of PRRs often leads to the assembly of intricate signaling complexes that recruit a variety of effectors, primarily protein kinases and ubiquitin ligases that will maintain signal transduction to the appropriate transcription factors while the stimulus persists [3]. MyD88 is a common component downstream all TLRs, with the only exception of TLR3, as well as interleukin-1 receptor (IL-1R) signaling [4–6].

MyD88 consists of two modular domains that mediate interactions with itself and other structurally similar proteins. The C-terminal Toll/Interleukin-1 receptor (TIR) domain associates with the cytoplasmic TIR domains of TLR receptors, while the N-terminal Death domain (DD) recruits downstream effectors, such as the IRAK4 kinase, through analogous interactions [2, 7]. Preceding the DD there is a short, 20-amino acid extension that remains structurally uncharacterized. The DD and TIR domains are separated by an intermediate region (INT), which is absent in the splicing variant MyD88-S [8]. Self-interaction of MyD88 is crucial for complex assembly; however, the precise stoichiometry and hierarchical organization of these assemblies is still a matter of debate [9].

It is well established that MyD88 is recruited to TLRs via interactions between their respective TIR domains [10], sometimes requiring accessory adaptors such as TIRAP, also known as MAL, in TLR4 signaling [11, 12]. MyD88 self-association is crucial for downstream assembly of the supramolecular organizing complex (SMOC) called myddosome [13, 14]. Recent findings indicate that shortly after MyD88 is primed at proto-myddosomes in contact with TLR receptors, larger dynamic barrel-like MyD88 structures detach from the receptor. These cytoplasmic SMOCs, considered proper myddosomes, recruit effector proteins to robustly transduce the signal. Eventually, such signalosomes are degraded by autophagy to terminate signaling [15].

It is not clear yet where the repository of MyD88 molecules lies in the cell prior to MyD88 activation. Ectopic expression of MyD88 in the absence of stimuli forms cytosolic aggregates that do not have a well-established localization [16, 17], although it is known that their formation does not rely on its TIR domain [18]. It has been proposed that MyD88 could form a preassembled complex in the cytoplasm ready to be recruited to TLR activation sites for seeding the active myddosome [19]. This is still controversial, as alternative evidence points towards the individual recruitment of MyD88 monomers after stimulation of either IL-1R [20] or TLR4 [13] for *de novo* proto-myddosome formation.

Mature myddosomes in PAMP-stimulated cells have not been found to associate directly with intracellular organelles in mammalian cells [15]. However, ectopic expression of MyD88 has been reported to lead to accumulation of the protein at a cytoplasmic spot, likely as a consequence of its autoaggregative features [17, 21–23]. Many aspects of MyD88 role in configuring the SMOCs remain still obscure. Heterologous expression in the budding yeast *Saccharomyces cerevisiae* serves as an “in vivo test tube” to study heterologous proteins in a eukaryotic intracellular environment devoid of their natural binding partners. We have previously shown that MyD88 localizes to endoplasmic reticulum-mitochondria encounter sites (ERMES) when heterologously expressed in yeast, and it is recruited to the plasma membrane upon TIRAP co-expression [24]. Here we take advantage of this model to study the contribution of MyD88 individual domains to self-assembly.

## Materials and methods

### Strains, media, and growth conditions

The *S. cerevisiae* YPH499 strain (*MATa ura3-52 lys2-801_amber ade2-101_ochre trp1-Δ63 his3-Δ200 leu2-Δ1*) was used in all experiments unless otherwise specified. The SEY5 strain (isogenic to BY4741; *dnm1∆::kanMX4*) and BY4741 (*MATa his3Δ1 leu2Δ0 met15Δ0 ura3Δ0*), used as a control, were employed to visualize cells lacking the Dnm1 protein. SEY5 was constructed by deleting the *DNM1* gene from YPH499 using a *kanMX4* cassette amplified from the pFA6-KanMX4 plasmid via homologous recombination. Deletion was confirmed through genomic PCR and fluorescence microscopy to assess mitochondrial phenotypes. Strain AF1 [isogenic to YPH499; *HO::GAL1-GFP(β1–9)-KanMX4*], which expresses a GFP variant lacking β10 and β11 strands, was used in tripartite GFP experiments. The *GAL1*-*GFP*(β1–9)-*KanMX4* cassette, amplified from the pGF-IVL794 plasmid, was integrated upstream of the *HO* start codon via homologous recombination. High-efficiency transformation of YPH499 with 45 µL of PCR product enabled genomic integration, which was confirmed by PCR using primers specific to *GFP*(β1–9) and the yeast genome. *Escherichia coli* DH5α was used for routine molecular biology.

Synthetic dextrose (SD) medium contained 2% glucose, 0.17% yeast nitrogen base without amino acids, 0.5% ammonium sulfate, and 0.12% synthetic amino acid drop-out mix, omitting specific components for plasmid selection. In synthetic galactose (SG) and synthetic raffinose (SR) media, glucose was replaced with 2% (w/v) galactose or 1.5% (w/v) raffinose, respectively. Media were prepared with deionized water (Elix Essential 10 system, Merck RRID: SCR_001287) and solidified with 2.4% (w/v) agar when necessary. All media were autoclaved at 121 °C for 20 min.

Protein induction driven by the *GAL1* promoter was performed by growing cells in SR medium to mid-exponential phase and refreshing cultures to an OD_600_ of 0.3 in SG medium lacking appropriate selection amino acids for 5 h unless otherwise noted. *E. coli* cultures were grown in LB medium supplemented with antibiotics at 100 µg/mL ampicillin, 50 µg/mL kanamycin, or 25 µg/mL chloramphenicol, as needed. Yeast and bacterial cultures were incubated at 30 °C and 37 °C, respectively, with shaking at 180 rpm.

### Plasmid construction

Standard molecular biology techniques were used following established protocols. Oligonucleotides and plasmids used are detailed in Supplementary Tables S1 and S2. Human cDNA for MyD88 and TIRAP was used for insert amplification. For Gateway cloning (Invitrogen RRID: SCR_005371), primers with *attB* sequences were designed using the manufacturer guidelines. PCR products were inserted into the pDONR221 vector using BP Clonase II (ThermoFisher Scientific, RRID: SCR_008452) to create Entry plasmids. Sequenced inserts were subcloned into *S. cerevisiae* Advanced Gateway Destination Vectors (Addgene Kit #1000000011, RRID: SCR_002037) [25] using LR Clonase II. BP and LR reactions followed manufacturer protocols, with modifications: clonase enzyme volume was reduced to 1 µL, and incubation extended to 18 h. Protein expression was validated by Western blotting. Empty vectors were generated by replacing the toxic *ccdB* gene with a polylinker amplified from pEG(KG) [26].

For MoClo cloning [27], reactions to assemble Part plasmids used the NEB Golden Gate Assembly Kit (*BsmB*I-v2; New England Biolabs RRID: SCR_013517), with additional steps at 60 °C for 10 min and 80 °C for 10 min. Final expression plasmids were assembled with sequenced Part plasmids using the *Bsa*I HF-v2 kit (New England Biolabs RRID: SCR_013517). Clones were verified via *Not*I digestion, and protein expression was confirmed by Western blotting. Coding sequences (CDS) cloned via the MoClo system were checked for *Bsa*I, *Bsm*BI, and *Not*I restriction sites, which were removed using two-step site-directed mutagenesis or the method described by Marillonnet and Grutzner [28]. Site-directed mutagenesis was performed using either by the two-step protocol described by Wang and Malcolm [29] or through overlapping PCR. In the first case, mutagenic oligonucleotides were designed following the guidelines of the QuikChange Site-Directed Mutagenesis kit (Agilent), using the *Pfu*Turbo DNA polymerase enzyme (Agilent Technologies, RRID: SCR_013575). Overlapping PCR, employed for both point mutations and the fusion of DNA fragments into a single amplicon, required a minimum overlapping region of 18 nucleotides and was performed with the Advantage HD Polymerase enzyme (Takara Bio Inc. RRID: SCR_021372).

### Spot growth assay

Yeast cultures were incubated overnight in SD medium. Then, each sample was diluted with sterile water in the first column of a 96-well plate to an OD_600_ of 0.5 in a final volume of 200 µL. Three tenfold serial dilutions were then prepared in the subsequent columns by transferring 20 µL from the first dilution into 180 µL of sterile water pre-filled in the 2nd, 3rd, and 4th columns. Finally, the dilutions were inoculated onto the surface of SD and SG agar plates using the Multi-blot VP 407AH replicator (V&P Scientific Inc., San Diego, CA, USA). The plates were incubated at 30 °C for 48–72 h.

### Immunodetection by Western blotting

Galactose-induced yeast cultures (20 mL) were collected by centrifugation at 2500 rpm and 4 °C during 3 min, and resuspended in 150 µL of lysis buffer (50 mM Tris-HCl pH 7.5; 10% glycerol; 1% Triton X-100; 0.1% NP-40; 0.1% SDS; 150 mM NaCl; 50 mM NaF; 50 mM β-glycerophosphate; 5 mM EDTA; 5 mM Na_2_P_2_O_7_; 1 mM Na_3_VO_4_) supplemented with 3 mM PMSF, 10 mM DTT, and a protease inhibitor cocktail (1 tablet/10 mL, ThermoFisher Scientific, RRID: SCR_008452). Mechanical lysis was performed using glass beads (ø = 0.75–1 mm) and a FastPrep^®^−24 system (RRID: SCR_018599) (two 30 s cycles at 5.5 m/s). After centrifugation at 13,000 rpm and 4 °C during 10 min, protein concentration in the supernatant was measured at 280 nm using a Beckman DU 640 spectrophotometer (RRID: SCR_008940). Samples were adjusted to the lowest protein concentration and mixed with 2×SDS-PAGE loading buffer (125 mM Tris-HCl pH 6.8; 25% glycerol; 250 mM DTT; 5% SDS; 0.2% bromophenol blue) in a 1:1 ratio and heated at 99 °C for 5 min.

Polyacrylamide gels were prepared with a 10% separating gel (10% acrylamide-bisacrylamide, 0.38 M Tris-HCl pH 8.8, 0.1% SDS, 0.05% ammonium persulfate [APS], 1.3 µL/mL tetramethylethylenediamine [TEMED]) and a 5% stacking gel (5% acrylamide-bisacrylamide, 0.25 M Tris-HCl pH 6.8, 0.1% SDS, 0.1% APS, 2 µL/mL TEMED). 10 µL of protein samples were loaded into the wells, and molecular weight markers (Abcam RRID: SCR_012931) were included. SDS-PAGE was run at 120 V using standard running buffer (25 mM Tris base; 192.6 mM glycine; 0.1% SDS).

After electrophoresis, proteins were transferred onto a 0.45 μm nitrocellulose membrane (Cytiva Life Sciences Amersham Protran, RRID: SCR_013566) using Mini Trans-Blot Cell tanks (Bio-Rad, RRID: SCR_008426) at 110 V for 1 h with transfer buffer (48 mM Tris base; 38.6 mM glycine; 0.037% SDS; 20% ethanol). The membrane was blocked with 5% skim milk in PBS for 1 h at room temperature with gentle agitation. Primary antibody incubation was performed overnight at 4 °C with agitation, using antibodies diluted in 0.1% PBS-T with 1% skim milk. Then, after five 5-min washes with PBS-T, the membrane was incubated for 1 h at room temperature in darkness with fluorophore-conjugated secondary antibodies diluted in the same buffer. Afterwards, washes were repeated.

Protein visualization was conducted with the ChemiDoc MP Imaging System (Bio-Rad, RRID: SCR_008426), and band intensity was quantified using Fiji (RRID: SCR_002285). The antibodies used in this study were as follows: Anti-GFP (JL-8) (Living Colors, Cat. No. 632380, Takara Bio Inc. RRID: SCR_021372, dilution 1:1000), Anti-FLAG (Sigma-Aldrich, Cat. No. F3165, RRID: SCR_008988, dilution 1:1000), Anti-G6PDH (Sigma-Aldrich, Cat. No. A9521, RRID: SCR_008988, dilution 1:50,000), IRDye 800LT Goat anti-Mouse IgG (LI-COR Biosciences, Cat. No. 926-32210, RRID: SCR_013430, dilution 1:50,000), and IRDye 680CW Goat anti-Rabbit IgG (LI-COR Biosciences, Cat. No. 926-32211, RRID: SCR_013430, dilution 1:50,000).

### Microscopy techniques and image processing

For in vivo microscopy, galactose-induced cultures were harvested by centrifugation at 3000 rpm for 1 min. Differential interference contrast (DIC) and fluorescence microscopy were performed using either an Eclipse TE2000U inverted fluorescence microscope (Nikon, RRID: SCR_023161) or a Leica DMi8 automated inverted microscope (RRID: SCR_026672). In the former setup, digital images were captured using an Orca C4742-95-12ER camera (Hamamatsu Photonics, RRID: SCR_017105) mounted on the microscope and processed with HCImage software (Hamamatsu, RRID: SCR_015041). In the latter, images were processed with LAS X software (Leica, RRID: SCR_013673), enabling multidimensional image acquisition (multichannel and time-lapse) as well as 3D reconstructions, animations, and projections. Images were analyzed using ImageJ (RRID: SCR_003070) and Adobe Photoshop (RRID: SCR_014199).

### Flow cytometry

After 5 h of galactose induction, 1 mL of cell culture was harvested. Flow cytometry experiments were performed using a BD FACScan Scan (RRID: SCR_019596) equipped with a 488 nm excitation laser and emission filters with a bandwidth of 530/30 (FL1), 585/42 (FL2), and 670 (FL3) nm. In all cases, at least 10,000 cells per sample were analyzed. Data were processed using FlowJo (RRID: SCR_008520) software.

### GFP-tripartite system

Based on the work conducted in J.W. Thorner’s laboratory to adapt the tripartite GFP system in *S. cerevisiae* [30], two modifications were introduced to tailor it for this study. The first modification involved generating a yeast strain with the coding sequence for the incomplete GFP barrel, GFP(β1–9), integrated into the yeast genome. This was achieved by amplifying the *GAL1*-*GFP*(β1–9)-*kanMX4* cassette from the pGF-IVL794 plasmid via PCR. Using homologous recombination, the cassette was integrated upstream of the *HO* gene start codon. High-efficiency transformation of the YPH499 strain with 45 µL of unpurified PCR product enabled genomic integration, followed by selection on YPD medium supplemented with G418 (300 µg/mL). Successful integration in the AF1 strain was confirmed by PCR using primers specific for GFP(β1–9) and the yeast genome. The second modification involved cloning the coding sequences (CDS) of proteins to test interactions using the MoClo system [27], resulting in two collections. In the first collection, the genes were fused to *GFP* β-strand 10 and inserted into a plasmid with the *URA3* marker. In the second collection, the genes were fused to GFP β-strand 11 and inserted into a plasmid with the *HIS3* marker. To allow N- or C-terminal fusions, both β-strands 10 and 11 were cloned into MoClo part plasmids 3a and 4a. Short linkers of 5 and 10 amino acids were used for β−10 and β−11 fusions, respectively. To facilitate protein expression detection via Western blotting, the proteins were tagged with 3xFLAG-6xHis on the terminus opposite to the β-strand. The CDSs of the target proteins were cloned into MoClo plasmid part 3b.

### AlphaFold2 predictions

Protein structural models and protein complex assemblies were generated using AlphaFold2 (RRID: SCR_025454) [31] for individual proteins and AlphaFold2-multimer [32] for multi-protein complexes. These tools were accessed via ColabFold versions 1.5.3-patch or 1.5.5 [33]. To predict structural models, the primary amino acid sequence of the target protein was entered in the “query sequence” field, omitting the stop codon and any non-amino acid characters. For protein complexes comprising two or more proteins, the individual sequences were inputted, separated by colons (:), without spaces. The structural predictions presented in this study were performed using ColabFold’s advanced settings, with “PDB100” selected as the template mode and either “3” or “6” recycles applied, depending on the sequence length. Upon completion, ColabFold generated five ranked structural models labeled as “rank_1,” “rank_2,” “rank_3,” “rank_4,” and “rank_5.” Only the top-ranked model (“rank_1”), determined by quality metrics, was used in this study. The resulting PDB files were visualized and analyzed using UCSF ChimeraX (RRID: SCR_015872).

### Statistical analysis

Data analysis was performed using Microsoft Excel (RRID: SCR_016137) and IBM SPSS Statistics (RRID: SCR_016479), while graphical representations were generated using Origin (RRID: SCR_014212). Normality of the data was first evaluated with the Shapiro-Wilk test. In all experiments conducted in this study, the null hypothesis (H0) was retained. Accordingly, a two-tailed unpaired Student’s t-test was used for comparisons between two groups, whereas comparisons among more than two groups were performed using a One-Way ANOVA followed by Tukey’s honestly significant difference (HSD) post hoc test. Asterisks (*, **, ***) in the figures denote p-values of < 0.05, < 0.01, and < 0.001, respectively. All experiments were carried out as biological triplicates using different clones, with data presented as mean ± standard deviation, including error bars.

## Results

### The MyD88 death domain is sufficient for localization to yeast ERMES

We previously reported that the human MyD88 adaptor forms cytosolic spots that localize to ER-mitochondria membrane junctions when expressed in *S. cerevisiae* [24]. Given the controversy surrounding the subcellular localization of MyD88 in mammalian cells prior to TLR stimulation [18, 34], we aimed to further investigate this aspect in yeast. To this end, we generated a series of MyD88 truncations fused to fluorescent proteins to assess the contribution of its individual domains to this localization pattern.

**Fig. 1:**
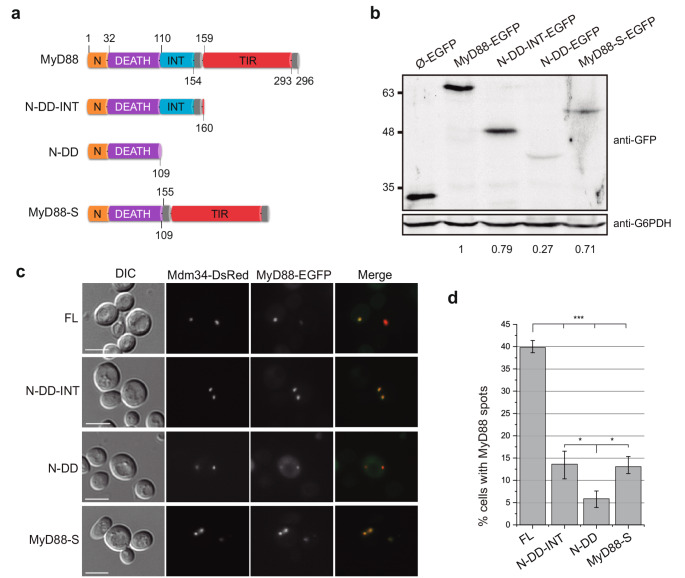
The absence of the INT and/or TIR domains reduces MyD88 stability in yeast while preserving ERMES localization. (**a**) Schematic representation of the different MyD88 variants: the wild type protein (MyD88), a version lacking the TIR domain (N-DD-INT), one lacking both the TIR and INT domains (N-DD), and the splicing variant naturally occurring in human cells, lacking only the INT domain (MyD88-S). Domain color coding: orange for the N-terminal region, purple for the Death domain, blue for the INT domain and red for the TIR domain. (**b**) Immunoblot on cell extracts from the YPH499 strain transformed with plasmids pAG425-Ø-EGFP, pAG425-MyD88-EGFP, pAG425-MyD88-N-DD-INT-EGFP, pAG425-MyD88-N-DD-EGFP, or pAG425-MyD88-S-EGFP. Heterologous proteins were detected using an anti-GFP antibody, and the G6PDH protein was used as a loading control. The quantitative data shown represent the ratio of each band’s intensity relative to the loading control, normalized to wild-type MyD88. (**c**) Differential Interference Contrast (DIC) and fluorescence microscopy images of YPH499 cells co-transformed with plasmids pAG424-Mdm34-DsRed and pAG425-MyD88-EGFP, pAG425-MyD88-N-DD-INT-EGFP, pAG425-MyD88-N-DD-EGFP, or pAG425-MyD88-S-EGFP. The experiment was performed as a biological triplicate, and representative images are shown. Scale bar: 5 μm. (**d**) Bar graph showing the percentage of cells displaying detectable green fluorescence corresponding to MyD88 or its variants. Excitation conditions were identical for all four samples, and brightness and contrast were varied equally in all images. The experiment was performed as a biological triplicate and > 50 cells of each transformant were analyzed. Error bars represent the SD. Asterisks indicate a p value < 0.001 (***) and < 0.05 (*), calculated using Tukey’s HSD test

Firstly, we designed truncated MyD88 variants that retained the Death domain (DD) in all cases, as this domain is essential for myddosome oligomerization [19]. Three variants were generated (Fig. 1a): one lacking the TIR domain and spanning amino acids 1-160 (N-DD-INT), another additionally lacking the INT domain and comprising amino acids 1-109 (N-DD), and the MyD88-S splicing variant, which lacks the INT domain [8]. Overproduction of these MyD88 versions, like that of the WT protein, did not affect yeast growth, as determined by a drop growth assay (Fig. S1a). As shown in Fig. 1b, immunoblot analysis revealed variations in the expression levels of the different truncated MyD88-EGFP versions. The N-DD and MyD88-S variants exhibited lower protein levels compared to the N-DD-INT and WT versions, with a particularly pronounced reduction in N-DD. This suggests that the TIR and, especially, the INT domain contribute to protein stability.

The elimination of the TIR and/or INT domains of MyD88 did not modify the subcellular localization pattern, which remained identical to that of the full-length protein, colocalizing with the ERMES component Mdm34 tagged with DsRed (Fig. 1c). However, consistently with the observed loss of protein stability, a significant reduction in the number of cells displaying MyD88 spots was detected (Fig. 1d).

### Deletion of residues 1–20 upstream MyD88 Death domain shifts its aggregation from spots to filaments

**Fig. 2:**
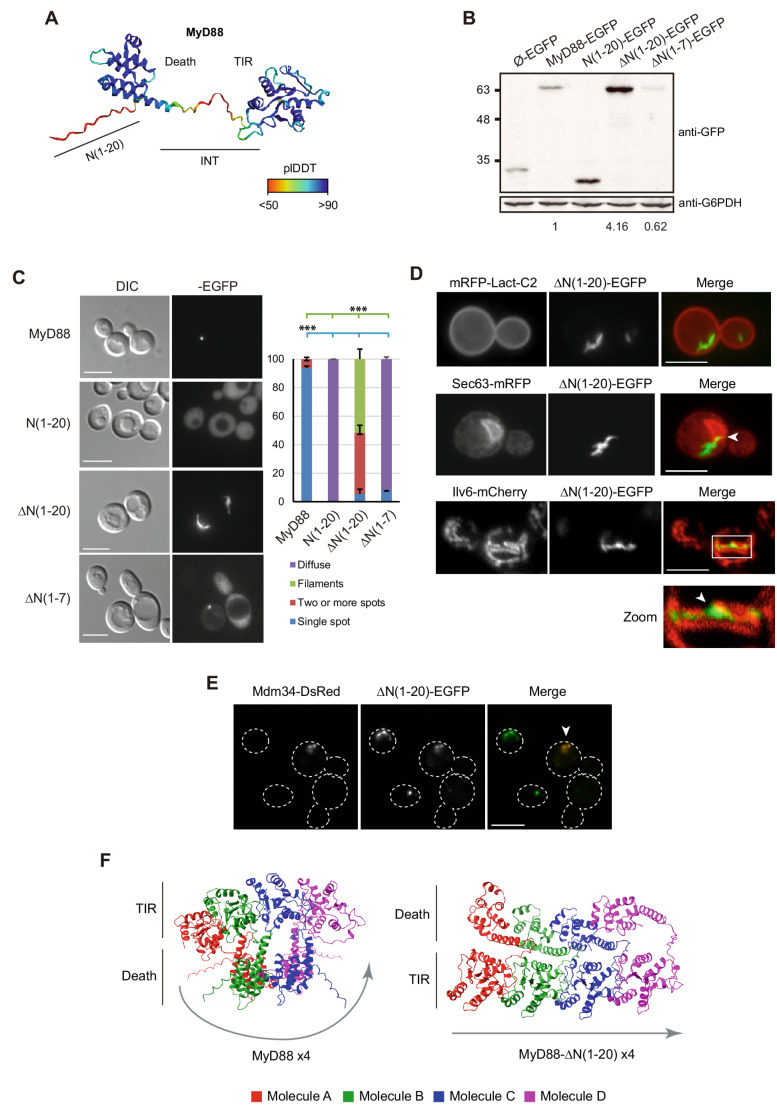
The first 20 amino acids at the N-terminus of human MyD88 influence its stability and homopolymerization pattern. (**a**) Predicted three-dimensional structure of MyD88 generated using ColabFold v1.5.3-patch. Amino acid residues are colored according to the confidence of the prediction, based on the plDDT (predicted local distance difference test) score. The model has an average plDDT of 83.4 and a pTM-score of 0.521. A total of 6 recycling steps were applied during the prediction. (**b**) Immunoblot of cell extracts from YPH499 strain transformed with plasmids pAG425-Ø-EGFP, pAG425-MyD88-EGFP, pAG425-MyD88-N1-20-EGFP, pAG425-MyD88-∆N1-20-EGFP or pAG425-MyD88-∆N1-7-EGFP. Heterologous proteins were detected using an anti-GFP antibody, with G6PDH as the loading control. Quantification shows the ratio of each band intensity relative to the loading control, normalized to wild-type MyD88-EGFP. (**c**) DIC and fluorescence microscopy images of YPH499 cells transformed with the same plasmids as in panel (**b**). Representative fields are shown. A graph is shown displaying the percentage of cells with distinct MyD88 subcellular localizations, as noted. Error bars represent the SD from three different biological replicates (*n*≈100 for each transformant clone). Statistical significance of cells with a single spot on WT MyD88 vs. all truncations (blue), as well as cells with filamentous structures in MyD88-∆N1-20 vs. the rest (green) are indicated with a p-value < 0.001 (***), according to Student t-test analyses. (**d**) Fluorescence microscopy images of YPH499 cells co-transformed with plasmids pGPD416-mCherry-Lact-C2 and pAG425-MyD88-∆N1-20-EGFP (top panels); pSM1959-Sect. 63-mRFP and pAG426-MyD88-ΔN1-20-EGFP (middle); or YEplac112-Ilv6-mCherry and pAG425-MyD88-ΔN1-20-EGFP (bottom). (**e**) Fluorescence microscopy images of YPH499 cells co-transformed with pAG424-Mdm34-DsRed and pAG425-MyD88-ΔN1-20-EGFP. All microscopy experiments were performed with biological triplicates, and representative images are shown. Scale bars represent 5 μm. (**f**) Predicted three-dimensional structures of four MyD88 molecules (left panel) and four MyD88-∆N(1–20) molecules (right panel) generated with ColabFold v1.5.5. Each monomer is shown in a different color (red, green, blue, purple). The MyD88 × 4 model has an average plDDT of 66.6, a pTM-score of 0.333, and an ipTM-score of 0.254. On the other hand, the ∆N1-20 × 4 model has an average plDDT of 71.8, with the pTM-score and ipTM-score values of 0.437 and 0.376, respectively. Three recycling steps were applied for both models

The complete three-dimensional structure of MyD88 has not yet been solved experimentally. To gain further insight into its structure, we performed structural modeling of the full-length protein using ColabFold v1.5.3-patch [31]. In the prediction model shown in Fig. 2a, the Death and TIR domains are resolved with a high level of confidence (plDDT > 90, or < 90 and > 70). However, the N-terminal region, corresponding to the first 20 amino acids, as well as certain fragments of the INT domain, exhibit significantly lower confidence scores (plDDT < 70 and > 50, or < 50). This may reflect that these are disordered regions, less conserved in evolution as the Death and TIR domains. Indeed, a multiple sequence alignment of the MyD88 sequence from 25 metazoan species reveals that a reliable consensus sequence can only be established starting at position 20 in the human MyD88 sequence. Furthermore, we observed some correlation between the extension of the N-terminal region and the evolutionary branch within vertebrates: the extension is generally longer in mammals, intermediate in birds, reptiles, and amphibians, and shorter in fish (Fig. S2).

Considering this information, along with previous studies suggesting that the N-terminal region of MyD88 is responsible for its subcellular localization [18, 35], we expressed two truncated versions of MyD88 in yeast: one lacking the first 7 amino acids (MyD88-∆N1-7), corresponding to the shortest MyD88 sequences found in fish, and another lacking the whole 20 amino acid N-terminal extension (MyD88-∆N1-20). Additionally, the first 20 amino acids of MyD88 were cloned separately (N1-20) to assess whether this segment alone was sufficient to drive a specific subcellular localization pattern in yeast. Like WT MyD88, expression of these truncated variants did not cause toxicity in yeast (Fig. S1b). Remarkably, as shown in Fig. 2b, the MyD88-ΔN1-20 EGFP fusion was immunodetected from yeast lysates to a greater extent than that of full-length MyD88, whereas MyD88-∆N1-7 yielded a fainter band. Regarding subcellular localization, the ∆N1-20 variant predominantly formed cytoplasmic filaments instead of compact puncta in a significant proportion of cells (51.71 ± 7.06%), a behavior never observed for full-length MyD88, as well as cells with more than one fluorescent spot (42.77 ± 5.31) (Fig. 2c). In contrast, the ∆N1-7 variant significantly lost the characteristic punctate pattern (spots were only observed 8.46 ± 1.50% of the cells). The construction comprising only the first 20 amino acids of MyD88, N(1–20), presented a diffuse cytosolic pattern in 100% of the cells (Fig. 2c). These results indicate that such N-terminal extension itself is not responsible for the subcellular localization of MyD88 in yeast cells. Moreover, its complete removal appears to enhance its aggregation properties and its stability, whereas partial truncation has the opposite effect.

Next, we investigated whether the filamentous structures formed by the ∆N1-20 version of MyD88 were associated with any cellular membrane. To this end, we co-expressed it with fluorescent markers for the plasma membrane, ER and mitochondria (Fig. 2d). To assess plasma membrane association, we used the phosphatidylserine-binding domain Lact-C2 fused to mRFP (mRFP-Lact-C2), which labels the inner leaflet of the plasma membrane [36]. No association of MyD88-ΔN1-20 filaments with the plasma membrane was observed. For ER visualization, we employed the Sec63-mRFP marker. Although MyD88-∆N1-20 filaments did not co-localize with the ER, their ends frequently converged or were in close proximity to perinuclear ER regions (Fig. 2 d, arrowhead; see focal planes in Fig. S3). Co-localization analyses with the mitochondrial marker Ilv6-mCherry revealed that MyD88-ΔN1-20 filaments were often positioned near and longitudinally associated with yeast mitochondria. These findings suggest that MyD88-∆N1-20 filaments extend from ER-mitochondria contact sites, consistent with ERMES localization (see arrowhead in zoomed image in Fig. 2 d). Finally, co-expression of MyD88-ΔN1-20-EGFP with the Mdm34-DsRed marker confirmed co-localization with this ERMES component (Fig. 2e). Thus, the N-terminal 20-amino acid extension is dispensable for MyD88 attachment to ER-mitochondria contact sites.

Although our results suggest that the overlooked N-terminal extension of MyD88 seems to play an important role in its aggregation properties and its stability, AlphaFold2 did not predict significant structural differences between WT MyD88 and either its ∆N1-20 or ∆N1-7 versions (data not shown). However, interestingly, comparative structural modeling of oligomeric assemblies using AlphaFold2-multimer revealed notable differences between the predicted organization of four WT MyD88 molecules and four MyD88-∆N1-20 molecules. While MyD88 molecules appeared to nucleate around a single central point, MyD88-∆N1-20 molecules assembled in a filamentous fashion (Fig. 2f). According to the Predicted Aligned Error (PAE) graphs obtained (Fig. S4), the relative positioning of the TIR domains within these complexes is more precisely defined than that of the Death domains. This differential structural prediction of the intermolecular interactions may explain the distinct aggregation patterns observed in yeast, with WT MyD88 forming compact puncta and MyD88-∆N1-20 assembling into filaments.

### Dynamin-like protein Dnm1 is necessary for proper MyD88 expression in yeast

To obtain genetic evidence supporting the association of MyD88 with ER-mitochondria contact sites, we attempted to construct yeast strains lacking individual ERMES components, specifically knocking out *MDM34*,* MMM1*,* MDM10*, or *MDM12* genes. However, as previously reported [37], these deletions result in severe defects in mitochondrial DNA maintenance and inheritance. Consequently, the mutants acquired a *petite* phenotype, as has also been described by other authors [38]. In our hands, these mutants exhibited high clonal variability, preventing the consistent expression of the heterologous protein and yielding inconclusive results. Therefore, we opted to use a *dnm1*Δ deletion mutant, which lacks dynamin 1 (Dnm1) [39] and exhibits a less severe mitochondrial phenotype. Dnm1 plays a key role in mitochondrial fission, and its deletion leads to collapsed mitochondria [40]. Despite this alteration, ERMES integrity remains intact in *dnm1*Δ mutants, although the number of ER-mitochondria contact sites is reduced. Notably, mitochondrial inheritance is more efficient in *dnm1*Δ cells compared to ERMES mutants [41].

**Fig. 3:**
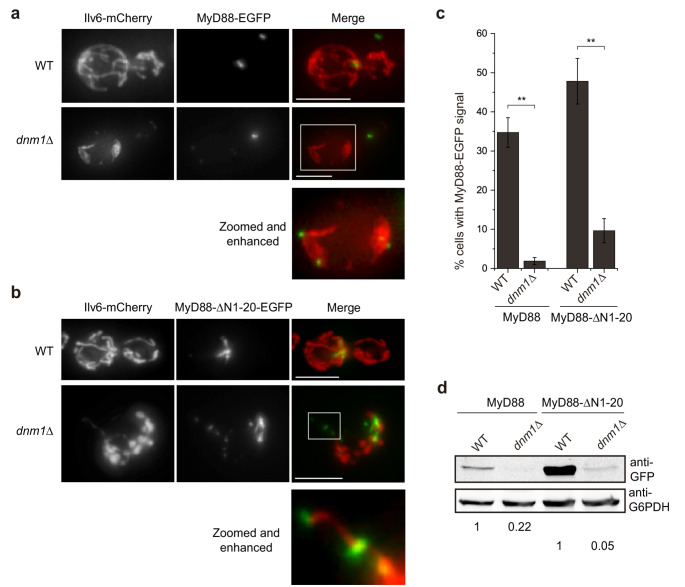
The expression of MyD88 and MyD88-∆N1-20 is reduced in a *dnm1*∆ mutant. (**a**, **b**) Maximum projection of Z-stack fluorescence microscopy images of BY4741 wild type (WT) cells (upper panels) and SEY5 (*dnm1*∆) mutant cells (lower panels), co-transformed with YEplac112-Ilv6-mCherry and pAG425-MyD88-EGFP (**a**) or pAG425-MyD88-ΔN1-20-EGFP (**b**). Scale bar corresponds to 5 μm. (**c**) Bar graph showing the percentage of cells with detectable MyD88-EGFP or MyD88-ΔN1-20-EGFP fluorescence in WT and *dnm1*Δ strains, as observed by fluorescence microscopy. The experiment was performed as a biological triplicate, with > 100 cells analyzed per transformant. Error bars represent SD. Asterisks indicate a p value < 0.01 (**), calculated using Student’s t test. (**d**). Immunoblot of cell extracts from BY4741 and isogenic *dnm1*∆ strains transformed with pAG425-MyD88-EGFP. MyD88 variants were detected using an anti-GFP antibody, with anti-G6PDH serving as a loading control. Quantification shows the ratio of each band’s intensity relative to the loading control, normalized to the corresponding construction in the WT strain

A strong loss of the amounts of MyD88 and MyD88-∆N1-20 was observed in the *dnm1*∆ mutant, as both proteins were detected at much lower levels compared to the WT strain, as assessed by fluorescence microscopy (Figs. 3a-c) and by Western blotting (Fig. 3 d). However, their subcellular localization pattern did not vary substantially in the *dnm1*∆ mutant as compared to the WT strain. Although the mitochondrial network was completely unstructured, in the few cells where MyD88-EGFP fluorescence was still detectable (approximately 2% for WT MyD88 and 10% for the truncated version; see Fig. 3c), full-length MyD88 remained adjacent to mitochondria (Fig. 3a), and MyD88-ΔN1-20 filaments also remained associated with them (Fig. 3b). These observations suggest that the ERMES continues to serve as a nucleation point for both constructs, but Dnm1 appears to be essential for maintaining levels of both MyD88 and MyD88-ΔN1-20 in the yeast cell.

### Linking the N-terminal localization domain of TIRAP to MyD88 shifts its affinity from plasma membrane to mitochondria

**Fig. 4:**
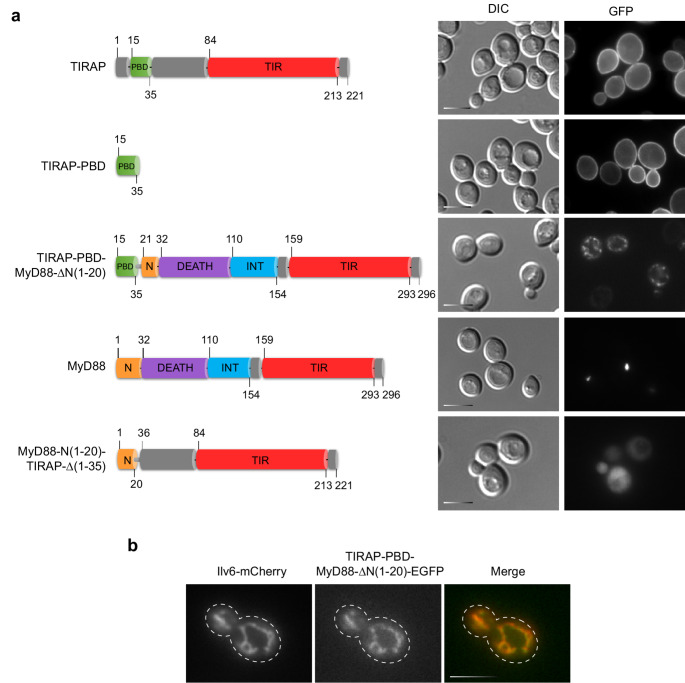
Subcellular localization of TIRAP-MyD88 chimeric proteins. (**a**) Fluorescence microscopy images of YPH499 cells transformed with the plasmids pAG425-Ø-EGFP, pAG425-TIRAP-EGFP, pAG425-TIRAP-PBD-EGFP, pAG425-TIRAP-PBD-MyD88-∆N1-20-EGFP, pAG425-MyD88-EGFP, or pAG425-MyD88-N1-20-TIRAP-∆1-35-EGFP, as indicated in the schematic diagram on the left. Protein fragments in the chimeric constructs are linked through a glycine-alanine bridge (Gly-Ala×5). (**b**) Fluorescence microscopy images of YPH499 cells co-transformed with YEplac112-Ilv6-mCherry and pAG425-TIRAP-PBD-MyD88-∆N1-20-EGFP. Scale bar corresponds to 5 μm. All experiments were performed in biological triplicate, and representative images are shown

The TIRAP adaptor consists of two main domains: the PtdIns(4,5)P_2_-binding domain (PBD), spanning amino acids 15–35 and responsible for plasma membrane localization [17], and the TIR domain, which interacts with MyD88 [42] (Fig. 4a). *S. cerevisiae* is a reference model organism in the study of phosphoinositide-dependent signaling, as its plasma membrane, like that of mammalian cells, is enriched in PtdIns(4,5)P₂ [43]. We have previously reported that human TIRAP is localized to the plasma membrane when expressed in yeast [24].

To investigate whether MyD88 localization could be redirected from the ERMES to the plasma membrane, we replaced its N-terminal extension with the PDB signal of TIRAP. As a control, we expressed a TIRAP-EGFP fusion that localized at the plasma membrane (Fig. 4a). In contrast to our previous observations reporting non-toxicity of TIRAP in yeast with another expression vector [24], yeast cells expressing TIRAP from the pAG425 plasmid exhibited moderate growth inhibition (Fig. S1c), likely due to the higher expression levels achieved (Fig. S5). The PBD domain alone was sufficient to drive EGFP to the plasma membrane (Fig. 4a) in 100% of the cells. However, the chimeric construct in which the first 20 amino acids of MyD88 were replaced by the PBD signal (TIRAP-PBD-MyD88-∆N1-20) did not decorate the plasma membrane. Instead, it was found in 96.26 ± 0.45% of the cells located in cytoplasmic organelles (Fig. 4a) that, upon co-expression with the Ilv6-mCherry marker, were identified as mitochondria (Fig. 4b). This chimera was expressed with a lower efficiency than WT MyD88 (Fig. S5), indicating that the addition of the TIRAP-PBD N-terminal extension was deleterious for MyD88 self-assembly.

Conversely, replacing the PDB domain of TIRAP with the N-terminal extension of MyD88 (MyD88-N1-20-TIRAP-Δ1–35) abolished its plasma membrane localization, resulting in a diffuse cytoplasmic distribution (Fig. 4a) in 98.55 ± 0.42% of the cells. This further supports the idea that no specific spatial signals lie in the first 20 amino acids of MyD88. Unlike WT TIRAP, none of these fusions or truncations were toxic for the yeast cell (Fig. S1c). Overall, these data suggest that the affinity of the MyD88 Death domain for mitochondrial membranes is dominant over the characteristic ability of TIRAP’s PDB to bind PtdIns(4,5)P_2_ at the plasma membrane.

### The MyD88 TIR domain alone accumulates at lipid droplets, condensates mitochondria and is mildly toxic in *S. cerevisiae*

**Fig. 5:**
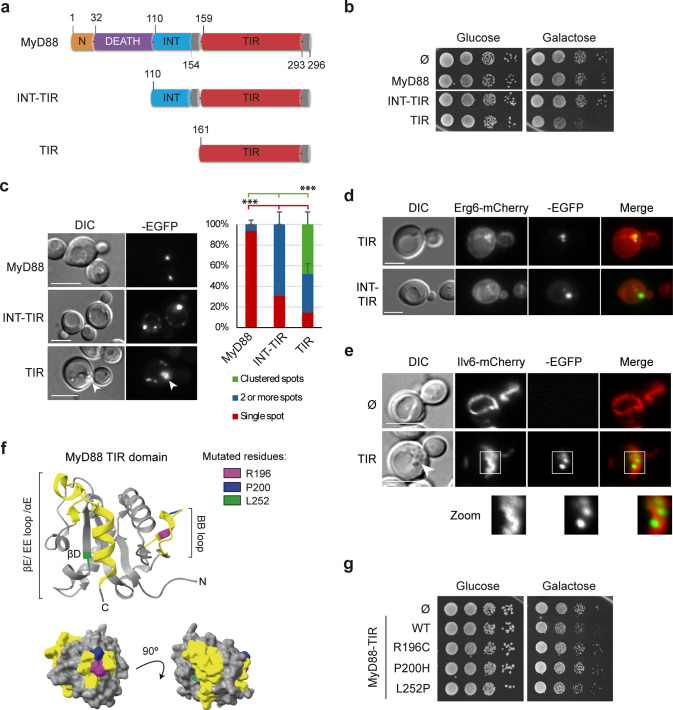
The TIR domain of MyD88 alone localizes to lipid droplets in yeast. (**a**) Schematic representation of MyD88 variants: wild type (MyD88), the INT and TIR domains (INT-TIR), and the isolated TIR domain (TIR). (**b**) Drop growth assay of YPH499 cells transformed with pAG425-Ø-EGFP, pAG425-MyD88-EGFP, pAG425-MyD88-INT-TIR-EGFP, or pAG425-MyD88-TIR-EGFP. (**c**) DIC and fluorescence microscopy images of YPH499 cells transformed with the plasmids listed in panel (**b**). The arrowhead indicates areas of enhanced refringency within the cells. Representative fields are shown. The graph at the right shows the percentage of cells with different kinds of MyD88 cytoplasmic spots, as noted. Error bars represent the SD from three different biological replicates (*n*≈50 for each transformant clone). Asterisks indicate a p-value < 0.001 (***), based on Student t-test analyses. (**d**) DIC and fluorescence microscopy images of YPH499 cells co-transformed with pGREG505-Erg6-mCherry and either pAG426-MyD88-TIR (top) or pAG426-MyD88-INT-TIR (bottom). (**e**) Expression of MyD88-TIR induces mitochondrial condensation in yeast. Representative DIC and fluorescence microscopy images of YPH499 cells transformed with the empty pAG425 plasmid or pAG425-MyD88-TIR. Scale bars correspond to 5 μm. (**f**) Ribbon and surface representations (top and bottom panels, respectively) of the three-dimensional structure of the MyD88 TIR domain (PDB: 4DOM). The BB loop (RDVLPGT), βE sheet, EE loop and αE helix are highlighted in yellow. Mutated residues are shown in color: magenta (R196C), blue (P200), and green (L252). (**g**) Loss-of-function mutations in the TIR domain eliminate toxicity in yeast. Drop growth assay of YPH499 cells transformed with plasmids pU323-Ø-Venus, pU323-MyD88-TIR-Venus, pU323-MyD88-TIR(R196C)-Venus, pU323-MyD88-TIR(P200H)-Venus, or pU323-MyD88-TIR(L252P)-Venus. Serial dilutions were plated on SD and SG-His synthetic media. All experiments were performed as biological triplicates; representative results are shown

After determining that the MyD88 TIR domain is dispensable for its localization at yeast ERMES, we decided to study this domain in isolation. To this end, we developed two new MyD88 variants expressed from the same vector: MyD88-INT-TIR, encompassing amino acids 110–296, and MyD88-TIR, spanning amino acids 161–296 (Fig. 5a). These truncated MyD88 variants, which lack the Death domain, were immunodetected to a much greater extent than the wild type protein, suggesting increased protein stability in yeast (Fig. S5b). Notably, expression of MyD88-TIR, but not MyD88-INT-TIR, induced a certain degree of toxicity to the yeast cell (Fig. 5b).

The subcellular localization pattern of these variants remained punctate but differed from that of the wild-type protein (Fig. 5c). In most cells, the INT-TIR-EGFP variant formed a single large dot per cell, accompanied by smaller puncta scattered throughout the cytosol. On the other hand, TIR-EGFP predominantly formed small clustered dots in regions exhibiting refractive changes under differential interference contrast (DIC) microscopy (see arrowhead in Fig. 5c), suggesting an association with lipid droplets (LDs). This hypothesis was confirmed by co-expressing MyD88-TIR-EGFP with Erg6-mCherry, a marker of both LDs and the ER, as LDs originate from the ER and often remain associated with it [44] (Figs. 5 d, upper panels, and S6a). Consistently, MyD88-TIR-EGFP spots were also found adjacent to perinuclear ER regions marked with Sec63-RFP (Fig. S6b). However, in the presence of the INT domain, INT-TIR-EGFP did not co-localize with Erg6-mCherry spots (Fig. 5 d, lower panels). The precise identity of the compartments where MyD88-INT-TIR-EGFP localized was not further investigated, but we ruled out their association with the JUNQ and IPOD proteostatic stress compartments through co-expression with the markers ChFP-Ubc9ts and Rnq1-mCherry [45] (Fig. S6c).

Higher expression levels of MyD88-TIR, achieved by using a different expression vector with a Venus fluorescent protein fusion (Fig. S7b), resulted in a clearer association with ER membranes, particularly at the cell periphery. In some cases, this also lead to filamentous structures spanning through the cytoplasm, suggesting self-association (Fig. S7d). This implies that expression levels (or perhaps the use of different GFP variants) affect MyD88-TIR self-association and its interaction with cellular membranes. Additionally, we overexpressed the TIR domain of TIRAP, deprived of its N-terminal plasma membrane localization signal, as a Venus fusion protein (Fig. S7c). As expected, this TIRAP-TIR-Venus fusion failed to localize to the plasma membrane and, like MyD88-TIR, localized to cytoplasmic spots coincident with areas of refringency changes in transmitted light microscopy (Fig. S7e). Taken together, these findings indicate that isolated TIR domains from human TLR signaling adaptors, when detached from their native localization signals, have an intrinsic affinity for yeast lipid droplets.

The observation of mitochondria using the Ilv6-mCherry marker in MyD88-TIR-EGFP-expressing cells revealed defects in mitochondrial morphogenesis. Mitochondria frequently appeared condensed near one of the cell poles, where the MyD88-TIR-EGFP signal was found in close proximity, overlapping with regions of refractive change in DIC microscopy (Fig. 5e, see arrowhead). These findings suggest that overexpression of the isolated MyD88 TIR domain disrupts ER-associated membranes, leading to mitochondrial dysfunction. This disruption may underlie the yeast growth inhibition observed specifically in this truncated MyD88 variant.

To determine whether TIR-TIR self-interaction was involved in the toxicity observed, we introduced point mutations in the asymmetric interaction interfaces of the MyD88 TIR domain, specifically in the BB loop and the βE/EE/αE loop, which are critical to homopolymer formation (Fig. 5f) [46, 47]. We generated the BB loop loss-of-function mutations R196C and P200H, the latter being equivalent to the P125H mutation within the TIR domain of TIRAP, which prevents TIR-TIR interactions [2, 7]. As a third variant, we introduced the oncogenic L252P mutation [48], which affects a residue located in the βD sheet, a region that is considerably less exposed (Fig. 5f). Loss-of-function mutants R196C and P200H no longer induced the slight growth inhibition observed with the wild-type TIR domain, whereas the gain-of-function mutant L252P exhibited a behavior similar to that of the wild type in this experimental setting (Fig. 5 g). Growth recovery in the loss-of-function mutants was not due to decreased protein stability, as immunoblot analyses confirmed that mutant protein levels were equivalent to those of the wild type version (Fig. S8a). Finally, fluorescence microscopy revealed that MyD88-TIR-Venus R196C, P200H, and L252P point mutants failed to form cytoplasmic filaments. Instead, all three mutants retained ER localization but tended to form compact spots (Fig. S8b).

### A sensitive fluorescence assay to test MyD88 interactions in vivo: the pathological L252P mutation limits self-association

**Fig. 6:**
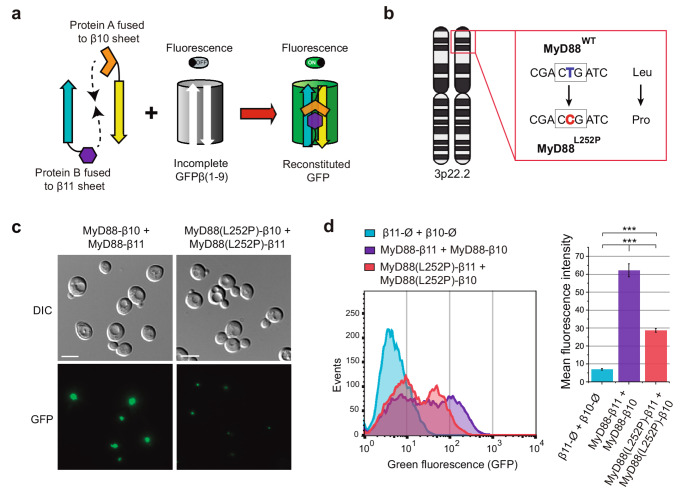
The oncogenic L252P mutation in MyD88 limits oligomer formation in yeast. (**a**) Schematic representation of the tripartite GFP assay. Two plasmids expressing the respective fusions of β10 and β11 GFP sheets to each of the proteins of interest are transformed into a yeast strain expressing a truncated incomplete β(1–9) form of GFP. Fluorescence is reconstituted only when the two proteins physically interact in space and time. (**b**) Diagram of human chromosome 3 showing the pathological point mutation that substitutes leucine 252 with proline in the MyD88 protein. (**c**) DIC and fluorescence microscopy images of AF1 strain cells transformed with plasmids pU316-3xFLAG-6xHis-MyD88-β10 and pU313-3xFLAG-6xHis-MyD88-β11 (left panels), or pU316-3xFLAG-6xHis-MyD88(L252P)-β10 and pU313-3xFLAG-6xHis-MyD88(L252P)-β11 (right panels). Experiments were performed as a biological triplicate, and representative images are shown. The scale bar corresponds to 5 μm. (**d**) Flow cytometry analysis. The left panel shows overlaid histograms where GFP fluorescence intensity is plotted on the X-axis and the number of events on the Y-axis. A representative result of a biological triplicate is shown. The right panel presents a bar graph of the mean fluorescence intensity of each sample. Error bars represent the SD. Statistical significance was determined using Tukey’s HSD test (***p < 0.001)

Accumulation of MyD88 at the ERMES or other cytoplasmic membranes could reflect accumulation of the misfolded heterologous protein. To rule out this possibility, we aimed to develop a yeast-based system capable of quantitatively assessing physiological MyD88 self-interactions. For this purpose, we used the tripartite GFP system [49] adapted to yeast [30]. Briefly, this system relies on the fusion of GFP β10 and β11 sheets to two putative interacting proteins, enabling the reconstitution of GFP over an incomplete β(1–9) GFP barrel upon interaction (Fig. 6a). We hypothesized that if properly folded MyD88 molecules were fused to both GFP β10 and β11 sheets, their polymerization would bring the sheets into proximity, leading to GFP reconstitution at the ERMES. To test this, we fused each β sheet to MyD88 at its C-terminus and studied self-interaction by these means. Control experiments using the individual fusions with plasmids expressing only the 3xFLAG-6xHIS tag fused to the corresponding β-sheet did not produce any fluorescence signal (data not shown), whereas the co-expression of MyD88-β10 and MyD88-β11 resulted in a robust fluorescent signal, indicating successful reconstitution of the tripartite GFP protein (Figs. 6c, d).

To evaluate the performance of the tripartite GFP assay, we examined the effect of the oncogenic L252P mutation on MyD88 self-interaction. This variant, reported as constitutively active, is the most prevalent mutation in Waldenström macroglobulinemia (Fig. 6c) [50]. Fluorescence microscopy revealed that this purported gain-of-function mutant formed a similar number of cytosolic puncta per cell as the wild type protein but with significantly lower fluorescence intensity (Fig. 6b). Western blot analyses confirmed that this reduction was not due to differences in protein expression levels (Fig. S9). As shown in Fig. 6d, this assay provides quantifiable data via flow cytometry. The difference in mean fluorescence intensity (MFI) between the wild type and L252P samples was statistically significant (Fig. 6 d, right panel). To sum-up, this yeast-based system represents a valuable tool for genetic and pharmacological screening of mutations or compounds that modulate MyD88 interactions.

## Discussion

MyD88 is the core myddosome adaptor operating in most TLR signaling pathways, where it self-interacts into higher-order structures in which both TIR and Death domains show additive effects [14]. Such structures evolve to interact with other TIR- or DD-domain containing proteins, thus amplifying signaling events upon sustained cell stimulation. It has recently been demonstrated that the myddosome is responsible for the execution of the entire signal transduction downstream TLR activation by PAMPs [15]. TLR stimulation primes the formation of a proto-myddosome associated to the plasma or endosomal membranes where the stimulus is received. In this initial stage, TIR-TIR interactions rule, but upon sustained stimulation the myddosome is released from the priming site to a cytoplasmic location where it grows to conform a dynamic large complex, whose core is based on DD-DD interactions involving MyD88 and IRAK kinases [15]. The study of these interactions is essential for understanding innate immune signaling and could ultimately aid in the development of therapeutic strategies to modulate the immune response in pathological scenarios. Here, we use the heterologous yeast model, which naturally lacks signalosome-like structures or endogenous proteins bearing DD or TIR domains to assess the contribution of MyD88 domains to self-assembly and recognition of molecular clues in a eukaryotic intracellular landscape.

Heterologous expression of human MyD88 in yeast results in the formation of protein aggregates, a behavior also observed upon ectopic expression in mammalian cells [17, 21–23]. Through co-localization studies with Mmm1 and Mdm34 proteins, we previously demonstrated that MyD88 aggregates in yeast are associated with ER-mitochondria contact sites, known as ERMES. Additionally, we found that TIRAP and MyD88 can mutually recruit each other to their respective locations, the plasma membrane and the ERMES, suggesting that their TIR domains are properly folded and able to interact [24]. Here, we show that the TIR domain of MyD88 is dispensable for its localization at the yeast ERMES. Instead, this localization depends on its Death domain. Furthermore, Dnm1, a dynamin-like protein associated with ERMES that is involved in mitochondrial fission and dynamics and is homologous to mammalian DRP1, was found important for robust MyD88 accumulation at this spot. In macrophages, the myddosome does not seem to interact with ER or mitochondrial markers, as addressed by systematic co-localization and co-purification experiments [15]. Thus, the behavior of MyD88 in yeast is intriguing. It may be related to the MyD88 cellular reservoirs in the absence of TLR activation, but it also raises the question of whether myddosomes may transiently interact with intracellular membranes in higher cells. Other innate immunity-related pathways, such as cGAS-STING and the NLRP3 inflammasome, have been related to mitochondria-ER contact sites [51–53]. Our experiments in yeast suggest that self-assembly at ERMES is crucial for MyD88 stability, as the absence of Dnm1 led to a dramatic reduction in MyD88 levels. We hypothesize that a priming signal for Death domain-mediated MyD88 nucleation exists at the yeast ERMES microenvironment, perhaps related to the presence of particular lipid species. Further studies will be required to elucidate its nature.

On the other hand, deletion of its N-terminal 20 amino acids (MyD88-ΔN1-20) enhanced MyD88 self-aggregation and stability. Instead of forming compact punctate structures, this variant assembled into filamentous stretches. Cryo-electron microscopy analyses of the MyD88 Death domain have revealed its ability to oligomerize and form helically symmetric filaments [19, 54]. Additionally, in vitro assays have shown that full-length MyD88 assembles into polymeric structures composed of more than 100 monomeric units [14]. Given that the size of the MyD88 oligomer regulates the intensity of TLR/IL-1R-dependent cell signaling [13, 20], we speculate that its N-terminal extension may function as a negative regulator, limiting MyD88 tendency to form larger homopolymeric structures, perhaps prioritizing interaction with IRAK4. AlphaFold2-multimer predictions indicate that both the Death and TIR domains contribute to the homotypic interactions between MyD88 molecules, regardless of the presence or absence of the 20-amino acid N-terminal extension. Notably, in both scenarios, the relative positioning of the TIR domains was more precisely defined than that of the Death domain. These results, together with the observation that the TIR domain contributes to MyD88 stability in yeast, support the idea that both domains may play a role in the prion-like association behavior of MyD88 [14, 55].

The nature of TLR4 proto-myddosomes associated to the mammalian plasma membrane is relatively transient, disappearing from the cell surface in minutes after LPS stimulation [13, 15, 20]. However, assembled myddosomes have been detected up to several hours after stimulation [3, 15, 56]. Therefore, myddosomes seem to detach from membranes and stably continue signaling from other locations, uncoupled from TLR/IL-1R receptors. Loosening of heterotypic TIR-TIR interactions involving MyD88 and its partners at the proto-myddosome, while promoting homotypic ones, should be crucial for this switch. Here, we show that the TIR domain of MyD88 alone was recruited to ER membranes and yeast LDs, and, when overexpressed, assembled filaments, in a fashion observed for other TIR domain-containing proteins, such as bacterial TIR-domain containing proteins and TRAM [24, 57]. This proves that homotypic TIR-TIR MyD88 interactions can take place in vivo even in the absence of its Death domain, as reported to happen in vitro [14]. The association of both MyD88 and TIRAP TIR domains with yeast LDs is intriguing. We cannot discard that misfolded heterologous proteins may accumulate at these lipid reservoirs, but it is tempting to speculate that the TIR domain of MyD88 is prone to liquid-liquid phase separation (LLPS) phenomena, as described for other protein aggregates [58]. We show that the introduction of mutations R196C or P200H in the BB loop of MyD88 TIR eliminated its toxicity by overexpression, its effects on mitochondrial morphology, and its ability to assemble intracellular filaments. Even if these results derive from severe overproduction in a heterologous system, they prove that TIR-TIR interactions account for the assembly of higher-ordered structures that can interfere with the function of cellular organelles.

The Waldeström macroglobulinemia-associated L252P mutation (also known in literature as L265P due to readjustments of numbering in the major isoform) is thought to induce structural changes in MyD88 that mimic the active conformation of its TIR domain when engaged with receptor TIR domains, or the phosphorylated active state of the protein [59]. This structural shift enables IRAK4 recruitment and downstream signaling activation in the absence of an external stimulus [2]. Our tripartite GFP results suggest that, contrary to what could intuitively be expected for a gain-of-function mutant, the MyD88 L252P oligomer is significantly smaller than the wild-type oligomer. This aligns with previous findings by O’Carroll and coworkers, who, based on in vitro experiments and in silico predictions, proposed that the oncogenic potential of this mutant lies in its ability to form stable low-order oligomers even at low protein concentrations [14].

In sum, we have used yeast as a model system to study MyD88, a key molecular target for antitumoral, anti-inflammatory and immunomodulating therapies, aiming to identify novel aspects of its function. The model provides a tractable in vivo cellular context to study its self-association properties devoid of other signaling pathway components. Tools like the tripartite GFP assay developed in this study should prove valuable for assessing the impact of various loss- and gain-of-function mutations on MyD88 oligomerization. Additionally, this system can be readily adapted for high-throughput screening of MyD88 polymerization inhibitors based on a rapid and cost-effective loss-of-fluorescence readout.

## Supplementary Information

Below is the link to the electronic supplementary material.

Supplementary Material 1

## Data Availability

No genomic or big data sets have been generated in this research. Raw image data of biological replicates for growth assays, immunoblots and microscopy images are available upon request.
