# Supplementary material for: MyD88 polymerization and association to cellular membranes in a yeast heterologous model
Source: Cell Mol Life Sci. 2025 Jul 25;82(1):288. doi: 10.1007/s00018-025-05827-1 (PMC12297185; doi:10.1007/s00018-025-05827-1)

Cellular and Molecular Life Sciences

Supplementary material to:

MyD88 polymerization and association to cellular membranes in a yeast heterologous model

Elba del Val, Alejandro Fernández-Vega, María Molina, Víctor J. Cid*

Department of Microbiology and Parasitology, School of Pharmacy, Complutense University of Madrid, Pza. Ramón y Cajal s/n. 28040-Madrid. Spain.

* Correspondence to Víctor J. Cid, vicjcid@ucm.es

Supplementary Tables

**Table S1**. Oligonucleotides used in this work	
Name	Sequence
Cloning oligonucleotides	
MyD88-Nt-attB1	ggggacaagtttgtacaaaaaa gcaggcttcATGGCTGCAGG AGGTCCC
MyD88-Nt-attB2	ggggaccactttgtacaagaaag ctgggttTCAGGGCAGGGA CAAGGC
MyD88-Ct-attB1	ggggacaagtttgtacaaaaaa gcaggcttcaccATGGCTGCA GGAGGTCC
MyD88-Ct-attB2	ggggaccactttgtacaagaaag ctgggtgGGGCAGGGACAA GGCC
MyD88- ΔTIR-attB2	ggggaccactttgtacaagaaag ctgggtgACGCTCAGGCATA TGCC
MyD88-N-DD-attB2	ggggaccactttgtacaagaaag ctgggtgAATGCTGGGTCCC AGCTC
MyD88-20-attB2	ggggaccactttgtacaagaaag ctgggtgAAGGGAGGATGT GGAGG
MyD88-Δ20-attB1	ggggacaagtttgtacaaaaaa gcaggcttcaccATGCCCCTG GCTGCTCTCAAC
MyD88-INT-TIR-attB1	ggggacaagtttgtacaaaaaa gcaggcttcaccATGGAGGA GGATTGCCAAAAG
MyD88-TIR-attB1	ggggacaagtttgtacaaaaaa gcaggcttcaccATGTTCGAT GCCTTCATCTGC
TIRAP-Ct-attB1	ggggacaagtttgtacaaaaaa gcaggcttcaccATGGCATCA TCGACCTCCCT

TIRAP-Ct-attB2	ggggaccactttgtacaagaaag ctgggtgAAGTAGATCAGAT ACTGTAGC
TIRAP-15-35-attB1	ggggacaagtttgtacaaaaaa gcaggcttcaccATGAAGAA GCCTCTAGGCAAG
TIRAP-15-35-attB2	ggggaccactttgtacaagaaag ctgggtgCTTCTTGGGCTTC TTCAG
MyD88-MC-Fw	gcatcgtctcatcggtctcatATG GCTGCAGGAGGTCC
MyD88-MC-Rv	atgccgtctcaggtctcaggatcc GGGCAGGGACAAGGCC
MyD88-TIR-MC-Fw	gcatcgtctcatcggtctcatatg TTCGATGCCTTCATCTGC
MyD88-TIR-MC-Rv	atgccgtctcaggtctcaggatcc GGGCAGGGACAAGGCC
TIRAP-TIR-MC-Fw	GCATCGTCTCATCGGTCTC ATATGAAAGACTATGACG TCTGC
TIRAP-TIR-MC-Rv	ATGCCGTCTCAGGTCTCA GGATCCGACAGCTTCTTT GACTTG
Oligonucleotides for overlapping PCR	
MyD88-S-Fw	gacgtgctgctggagctgggacc cagcattCATATGCCTGAGC GTTTCG
MyD88-S-Rv	gatgaaggcatcgaaacgctcag gcatatgAATGCTGGGTCCC AGCTC
15-35-TP-MyD-Fw	ggagctggtgcaggcgctggagc cggtgccCCCCTGGCTGCTC TCAAC
15-35-TP-MyD88 MyD-Rv	ggcaccggctccagcgcctgcac cagctccCTTCTTGGGCTTC TTCAG
20-MyD-TIRAP-Fw	ggagctggtgcaggcgctggagc cggtgccAGGCCCAACTCC CCAG
20-MyD-TIRAP-Rv	ggcaccggctccagcgcctgcac cagctccAAGGGAGGATGT GGAGG
TIRAP-MC-Fw	gcatcgtctcatcggtctcatatg GCATCATCGACCTCCCTC
TIRAP-MC-Rv	atgccgtctcaggtctcaggatcc AAGTAGATCAGATACTGT AGC
TIRAP-MC-Fw2	ATGCCGTCTCAGGTCTCA GGATCCAAGTAGATCAGA TACTGTAGC
TIRAP-MC-Rv2	ATGCCGTCTCAGGGTGAC

	GTGACTGAGC
Oligonucleotides for mutagenic PCR	
MyD88-MC-mut-Fw	GGTCTGCGGCCCCGGTTT CCTCCACATCCTCCC
MyD88-MC-mut-Rv	GGGAGGATGTGGAGGAA ACCGGGGCCGCAGACC
TIRAP-MC-mut-Fw	GTGGCCGCCCAGGACCT GGTTTCCTACTTGGAAGG CAG
TIRAP-MC-mut-Rv	CTGCCTTCCAAGTAGGAA ACCAGGTCCTGGGCGGC CAC
MyD88-L93P-Fw	CCGACTGCTCGAGCTGCC TACCAAGCTGGGCCGCG
MyD88-L93P-Rv	CGCGGCCCAGCTTGGTAG GCAGCTCGAGCAGTCGG
MyD88-R196C-Fw	AGTTGTGTGTGTCTGACT GCGATGTCCTGCCTGGC
MyD88-R196C-Rv	GCCAGGCAGGACATCGC AGTCAGACACACACAACT
MyD88 LP-Up	CAGAAGCGACCGATCCCC ATCAAGTACAAG
MyD88 LP-Lo	GGGGATCGGTCGCTTCTG ATGGGCA

**Table S2**. Plasmids used in this work	
Name	Reference
pDONR221 plasmid and Destination plasmids from the Gateway collection	
pDONR221-ccdB	Invitrogen
pAG425GAL-ccdB-EGFP	Addgene #1000000011
pAG425GAL-EGFP-ccdB	Addgene #1000000011
pAG426GAL-ccdB-EGFP	Addgene #1000000011
pAG413GAL-EGFP-ccdB	Addgene #1000000011
Entry plasmids generated with the Gateway system	
pENTRY-MyD88-Nt	This work
pENTRY-MyD88-Ct	This work
pENTRY-MyD88-N-DD-INT	This work
pENTRY-MyD88-N-DD	This work
pENTRY-MyD88-S	This work
pENTRY-MyD88-N(1- 20)	This work
pENTRY-MyD88-ΔN(1-20)	This work
pENTRY-MyD88-INT-TIR	This work
pENTRY-MyD88-TIR	This work
pENTRY-TIRAP	This work
pENTRY-TIRAP-PBD	This work
pENTRY-TIRAP-PBD-MyD88-ΔN(1-20)	This work
pENTRY-MyD88-20-TIRAP-Δ(1-35)	This work
pENTRY-Ø	This work

pENTRY-Ø-Ct	This work
Expression plasmids generated with the Gateway system	
pAG425-EGFP-MyD88	This work
pAG425-MyD88-EGFP	This work
pAG425-N-DD-INT-EGFP	This work
pAG425-N-DD-EGFP	This work
pAG425-MyD88-S-EGFP	This work
pAG425-N(1-20)-EGFP	This work
pAG425-ΔN(1-20)-EGFP	This work
pAG425-INT-TIR-EGFP	This work
pAG425-TIR-EGFP	This work
pAG425-TIRAP-EGFP	This work
pAG425-TIRAP-PBD-EGFP	This work
pAG425-TIRAP-PBD-MyD88-ΔN(1-20)-EGFP	This work
pAG425-MyD88-N(1-20)-TIRAP-Δ(1-5)-EGFP	This work
pAG425-Ø-EGFP	This work
pAG426-MyD88-EGFP	This work
pAG426-Ø-EGFP	This work
Part plasmids of the MoClo collection	
pYTK001	Addgene #65108
pYTK002	Addgene #65109
pYTK009	Addgene #65116
pYTK030	Addgene #65137
pYTK040	Addgene #65147
pP3-MyD88	This work
pP3-MyD88-TIR	This work
pP3-MyD88-TIR(R196C)	This work
pP3-MyD88-TIR(P200H)	This work
pP3-MyD88-TIR(L252P)	This work
pP3-TIRAP	This work
pP3-TIRAP-TIR	This work
pP3-Ø	This work
pP3a-GFP-β10	This work
pP3a-GFP-β11	This work
pYTK058	This work
pP4a-GFP-β10	This work
pP4a-GFP-β11	This work
pYTK063	Addgene #65170
pYTK072	Addgene #65179
pYTK074	Addgene #65181
pYTK076	Addgene #65183
pYTK081	Addgene #65188
pYTK082	Addgene #65189
pYTK088	Addgene #65195
pYTK083	Addgene #65190
pYTK089	Addgene #65196

pYTK094	Addgene #65201
Final yeast expression plasmids generated with the MoClo system	
pU323-MyD88-venus	This work
pU323-MyD88-TIR-venus	This work
pU323-MyD88-TIR(R196C)-venus	This work
pU323-MyD88-TIR(P200H)-venus	This work
pU323-MyD88-TIR(L252P)-venus	This work
pU323-TIRAP-venus	This work
pU323-TIRAP-TIR-venus	This work
pU323-Ø-venus	This work
pU313-3xFLAG-6xHis-MyD88-β11	This work
pU313-β11-MyD88-3xFLAG-6xHis	This work
pU316-3xFLAG-6xHis-MyD88-β10	This work
pU316-β10-MyD88-3xFLAG-6xHis	This work
pU313-3xFLAG-6xHis-MyD88(L93P)-β11	This work
pU313-3xFLAG-6xHis-MyD88(L252P)-β11	This work
pU316-3xFLAG-6xHis-MyD88(L252P)-β10	This work
Plasmids with organelle marker genes and signalling pathway indicators	
pSM1959	Addgene #41837
YEplac112-Ilv6-mCherry	Fernández-Acero et al., 2019
pOB06	This work
pESC-LEU-chFP-Ubc9ts	Kaganovich et al., 2008
p425-GAL1-Rnq1-mCherry	Kaganovich et al., 2008
pGPD416-mCherry-Lact-C2	This work
pGREG505-Erg6-mCherry	This work
Other plasmids	
pGF-IVL794	Finnigan et al., 2016
pFA6-kanMX4	Oka et al., 1981

Supplementary Figure legends

Figure S1. Overproduction of diverse MyD88 versions in yeast does not impair cell growth.

(a) Drop growth assay of YPH499 cells transformed with pAG425-Ø-EGFP, pAG425-MyD88-EGFP, pAG425-MyD88-N-DD-INT-EGFP, pAG425-MyD88-N-DD-EGFP, or pAG425-MyD88-S-EGFP. Serial dilutions of the cultures were spotted onto SD- and SG-Leu$^-$ agar plates. Dilutions for MyD88-S-EGFP were performed on the same plate as the other samples. **(b)** Drop growth assay of YPH499 cells transformed with pAG425-MyD88-EGFP, pAG425-MyD88-N1-20-EGFP, pAG425-MyD88-ΔN1-20-EGFP, or pAG425-MyD88-ΔN1-7-EGFP. Serial dilutions were plated on SD- and SG-Leu$^-$ media. All samples were plated on the same culture plate. **(c)** Drop growth assay of YPH499 cells transformed with pAG425-Ø-EGFP, pAG425-TIRAP-EGFP, pAG425-TIRAP-PBD-EGFP, pAG425-TIRAP-PBD-MyD88-ΔN1-20-EGFP, pAG425-MyD88-EGFP, pAG425-MyD88-N1-20-EGFP, or pAG425-MyD88-N1-20-TIRAP-Δ1-35-EGFP. Serial dilutions were plated on SD- and SG-Leu$^-$ media. All samples were plated on the same culture plate.

Figure S2. The first 20 amino acids of human MyD88 are not evolutionarily conserved.

Multiple sequence alignment of MyD88 proteins from various animal species, performed using Clustal Omega (Sievers et al., 2011) and visualized with MView (RRID:SCR_024129). The first column indicates the species, the second shows the sequence coverage in the alignment (cov), and the third the percentage identity (pid) relative to the human reference sequence. Residues matching the reference sequence are highlighted in color according to their physicochemical properties: dark green for aromatic amino acids [tryptophan (W), histidine (H), tyrosine (Y) and phenylalanine F)], light green for hydrophobic [valine (V), leucine (L), isoleucine (I), alanine (A), methionine (M), proline (P) and glycine (G)], dark blue for negative polar [aspartate (D) and glutamate (E)], red for positive polars [lysine (K) and arginine (R)], purple for neutral polars [asparagine (N) and glutamine (Q)], light blue for amino acids with an alcohol group [serine (S) and threonine (T)] and yellow for cysteine (C). At the bottom of the figure, consensus lines are shown at identity thresholds of 100%, 90%, 80% and 70%, based on the aforementioned residue classes.

Figure S3. Fluorescence microscopy images of YPH499 cells co-transformed with pSM1959-Sec63-mRFP and pAG426-MyD88-ΔN1-20-EGFP (middle panels). Different focal planes are shown, as indicated, to better illustrate the spatial relationship between red and green fluorescence signals. This figure complements the middle panel of Fig. 2d. Scale bar: 5 µm.

Figure S4. AlphaFold2-multimer PAE (Predicted Aligned Error) plots for structural models of MyD88×4 (left) and MyD88-ΔN1-20×4 (right), predicting distinct oligomerization patterns for the wild type and ΔN1-20 versions of MyD88. The sequences of the four molecules are plotted along both axes in sequential order. Diagonal quadrants display the predicted alignment error within the individual molecules, while off-diagonal quadrants show the predicted alignment error between different molecules. Expected position error is measured in Å and is indicated by the color scale in the figure.

Figure S5. (a) Immunoblot of cell extracts from YPH499 strain transformed with pAG425-Ø-EGFP, pAG425-TIRAP-EGFP, pAG425-TIRAP-PBD-EGFP, pAG425-TIRAP-PBD-MyD88-ΔN1-20-EGFP, pAG425-MyD88-EGFP, pAG425-MyD88-N1-20-EGFP, or pAG425-MyD88-N1-20-TIRAP-Δ1-35-EGFP. **(b)** Immunoblot of cell extracts from YPH499 strain transformed with pAG425-Ø-EGFP, pAG425-MyD88-EGFP, pAG425-MyD88-INT-TIR-EGFP, or pAG425-MyD88-TIR-EGFP. In both panels, heterologous proteins were detected with an anti-GFP antibody, and G6PDH was used as a loading control. In panel B, the quantitative data represent the ratio of the intensity of each GFP signal relative to the loading control, normalized to the MyD88 signal.

Figure S6. Subcellular localization of the TIR and INT-TIR domains of MyD88 in yeast. (a) Fluorescence microscopy images of YPH499 cells co-transformed with pGREG505-Erg6-mCherry and pAG426-MyD88-TIR-EGFP. Three focal planes are shown, as indicated below the images. **(b)** DIC and fluorescence microscopy images of YPH499 cells co-transformed with pSM1959-Sec63-RFP, and either pAG426-MyD88-TIR (top) or pAG426-MyD88-INT-TIR (bottom). **(c)** DIC and fluorescence microscopy images of YPH499 cells co-transformed with pAG426-MyD88-INT-TIR and either pESC-LEU-chFP-Ubc9ts (top) or p425-GAL1-Rnq1-mCherry (bottom). In the chFP-Ubc9ts experiment, the temperature was shifted to 37 °C during the last 30 min of incubation. All experiments were performed in biological triplicates, and representative images are shown. Scale bars: 5 µm.

Figure S7. Study of MyD88-TIR-venus and derived point mutants in yeast. (a) The fusion of the MyD88 TIR domain to the venus fluorescent protein is moderately toxic in yeast, similarly to full-length TIRAP, but not its TIR domain alone. Drop growth assay of YPH499 cells transformed with pU323-Ø-venus, pU323-MyD88-Venus, pU323-MyD88-TIR-Venus, pU323-TIRAP-Venus, or pU323-TIRAP-TIR-Venus. Serial dilutions were spotted on SD- and SG-Leu synthetic media plates. **(b)** MyD88-TIR-Venus is expressed at higher than MyD88-TIR-EGFP. Immunoblot of cell extracts from YPH499 cells transformed with pAG425-MyD88-TIR-EGFP or pU3-MyD88-TIR-Venus. Heterologous proteins were detected using an anti-MyD88 antibody. Quantification represents the ratio of each band intensity relative to the loading control, normalized to MyD88-TIR-EGFP. **(c)** Immunoblot of cell extracts from yeast transformants as in (a). Heterologous fusion proteins were detected using an anti-GFP antibody. In (b) and C, G6PDH protein was immunodetected as a loading control. **(d)** Fluorescence microscopy images of different focal planes of YPH499 cells co-transformed with pSM1959-Sec63-RFP and pU323-MyD88-TIR-Venus. The experiment was performed as a biological triplicate, and representative images are shown. Cells exhibit cytoplasmic spots and filamentous structures of MyD88-TIR-Venus, along with peripheral signal likely corresponding to the cortical ER. **(e)** Fluorescence microscopy images of YPH499 cells co-transformed with a plasmid expressing the plasma membrane marker mRFP-Lact-C2 and either pU323-Ø-venus (top panels) or pU323-MyD88-TIR-Venus (bottom panels). Scale bars correspond to 5 µm.

Figure S8. Expression of MyD88-TIR-Venus point mutants in yeast. (a) Immunoblot of cell extracts from YPH499 cells transformed with plasmids pU323-Ø-Venus, pU323-MyD88-TIR-Venus, pU323-MyD88-TIR(R196C)-Venus, pU323-MyD88-TIR(P200H)-Venus, or pU323-MyD88-TIR(L252P)-Venus. Heterologous proteins were detected using an anti-GFP antibody; G6PDH protein was used as a loading control. To better resolve differences in protein expression, 8 µL of the protein extracts were loaded, and

the nitrocellulose membrane was incubated with the primary antibody for 1 h at room temperature. Quantification represents the ratio of each band intensity relative to the loading control, normalized to MyD88. **(b)** Fluorescence microscopy images of different focal planes of YPH499 cells co-transformed with pSM1959-Sec63-RFP and pU323-MyD88-TIR-Venus, pU323-MyD88-TIR(R196C)-Venus, pU323-MyD88-TIR(P200H)-Venus, or pU323-MyD88-TIR(L252P)-Venus, as indicated. The experiment was performed as a biological triplicate, and representative images are shown. Mutant proteins localize predominantly as peripheral signal (likely corresponding to the cortical ER) and cytoplasmic puncta, but lack the filamentous structures observed in wild-type (see Fig. S7D). Scale bars: 5 µm.

Figure S9. **Expression of MyD88 constructs for tripartite GFP.** Immunoblot of cell extracts from strain AF1 transformed with plasmids pU316-3xFLAG-6xHis-Ø-β10 and pU313-3xFLAG-6xHis-Ø-β11; pU316-3xFLAG-6xHis-Ø-β10 and pU313-3xFLAG-6xHis-MyD88-β11; pU316-3xFLAG-6xHis-MyD88-β10 and pU313-3xFLAG-6xHis-Ø-β11; pU316-3xFLAG-6xHis-MyD88(L252P)-β10 and pU313-3xFLAG-6xHis-Ø-β11; or pU316-3xFLAG-6xHis-Ø-β10 and pU313-3xFLAG-6xHis-MyD88(L252P)-β11. Heterologous proteins were detected with the anti-FLAG antibody. The G6PDH protein was immunodetected as a loading control.

del Val et al. Fig. S1

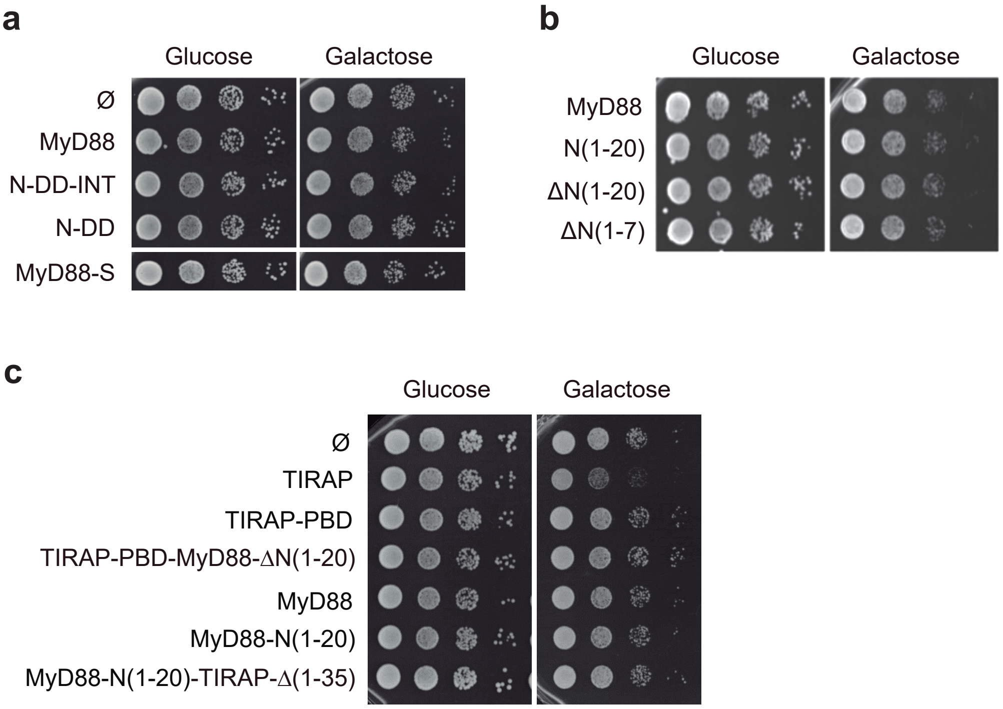

del Val et al. Fig. S2

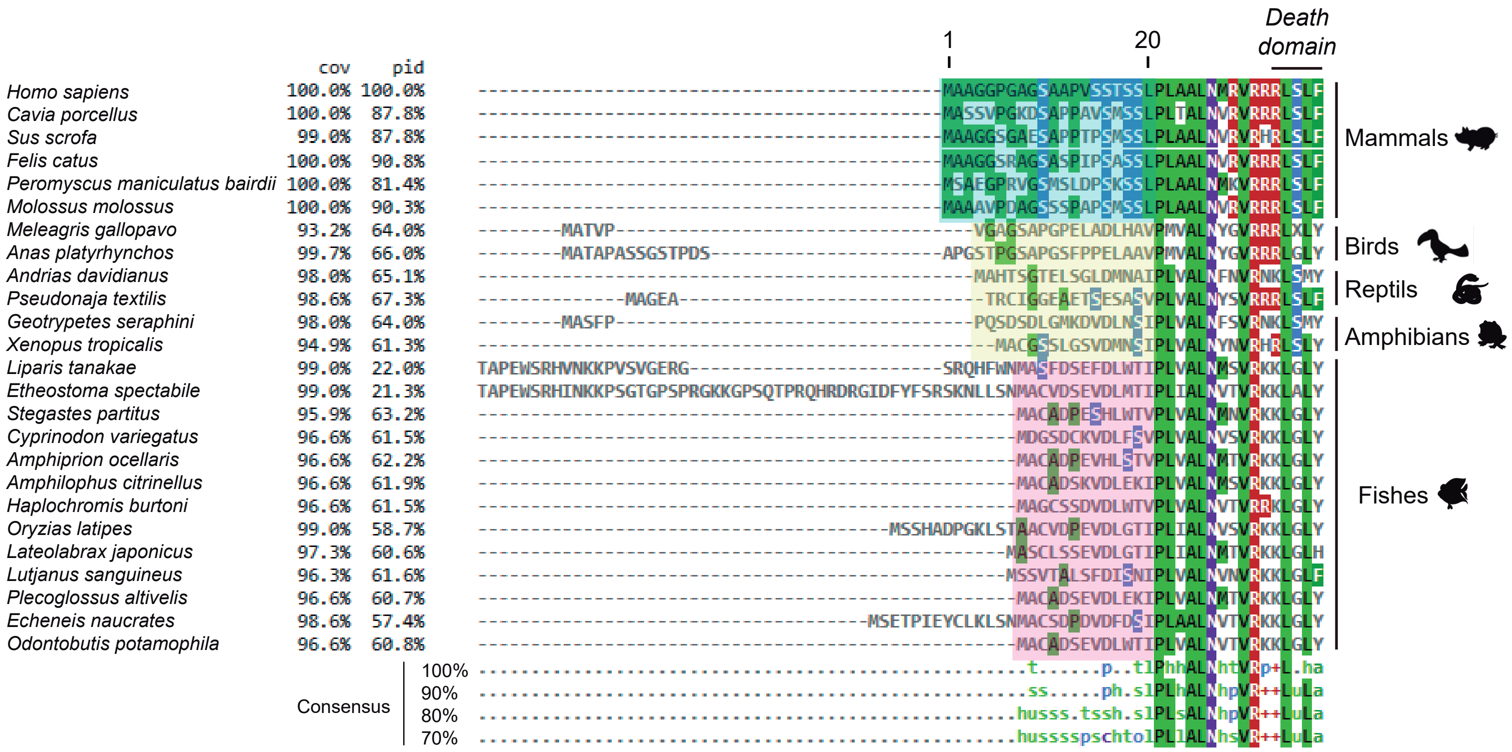

del Val et al. Fig. S3

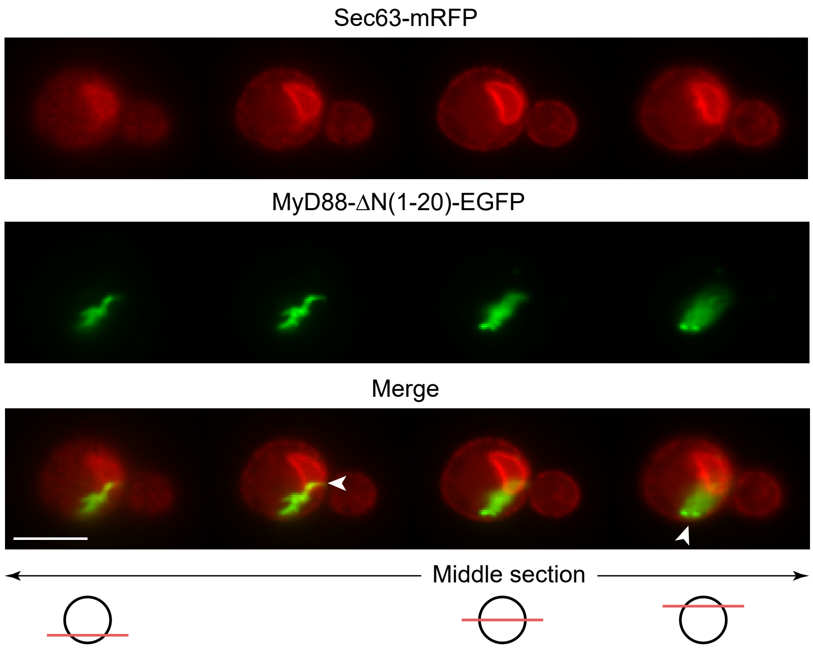

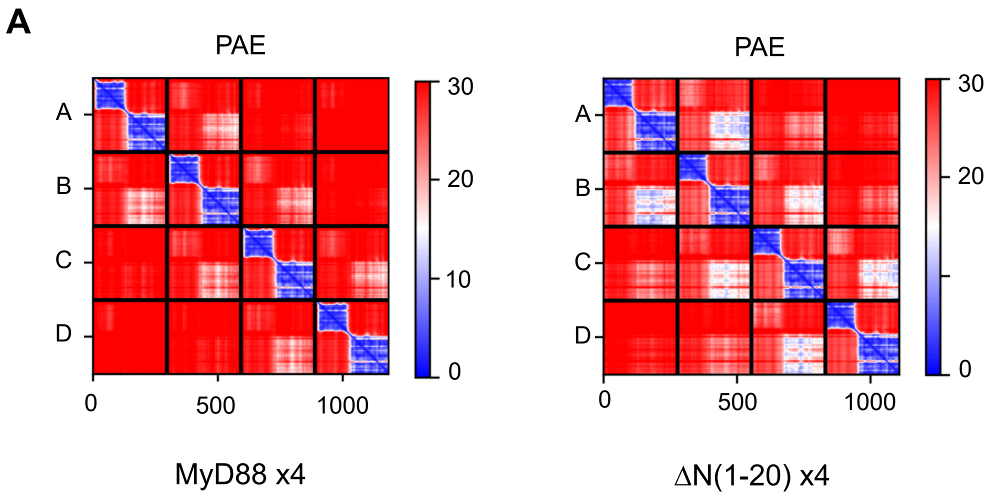
A
PAE
A
B
C
D
0
500
1000
30
20
10
0
MyD88 x4
PAE
A
B
C
D
0
500
1000
30
20
0
ΔN(1-20) x4

del Val et al. Fig. S5

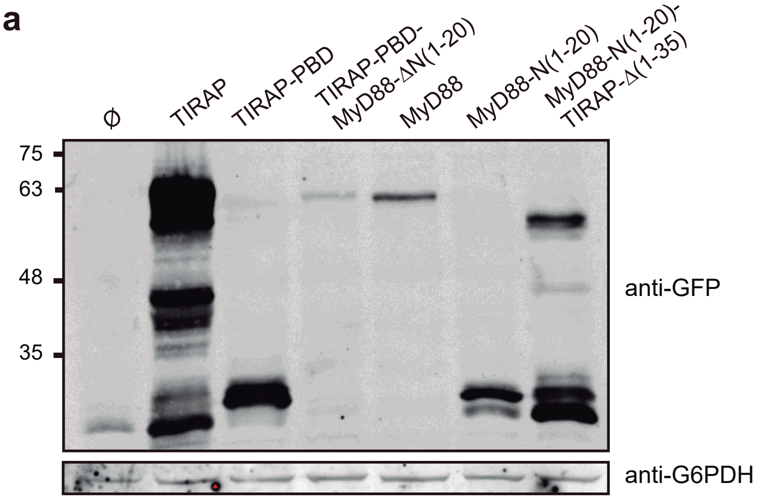

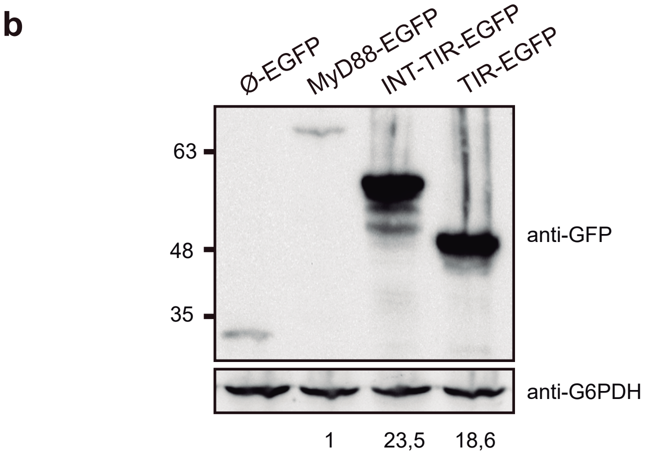

del Val et al. Fig. S6

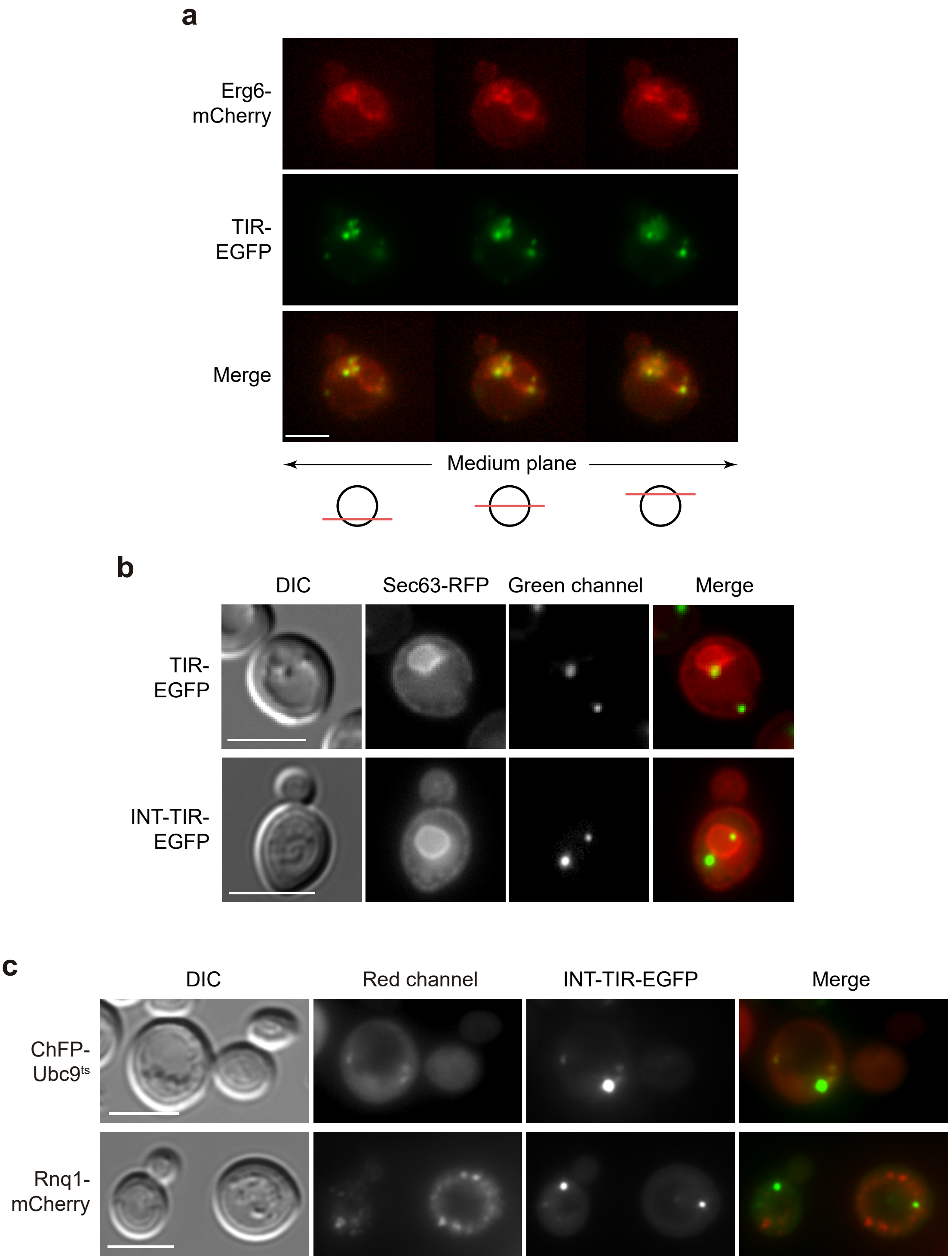

del Val et al. Fig. S7

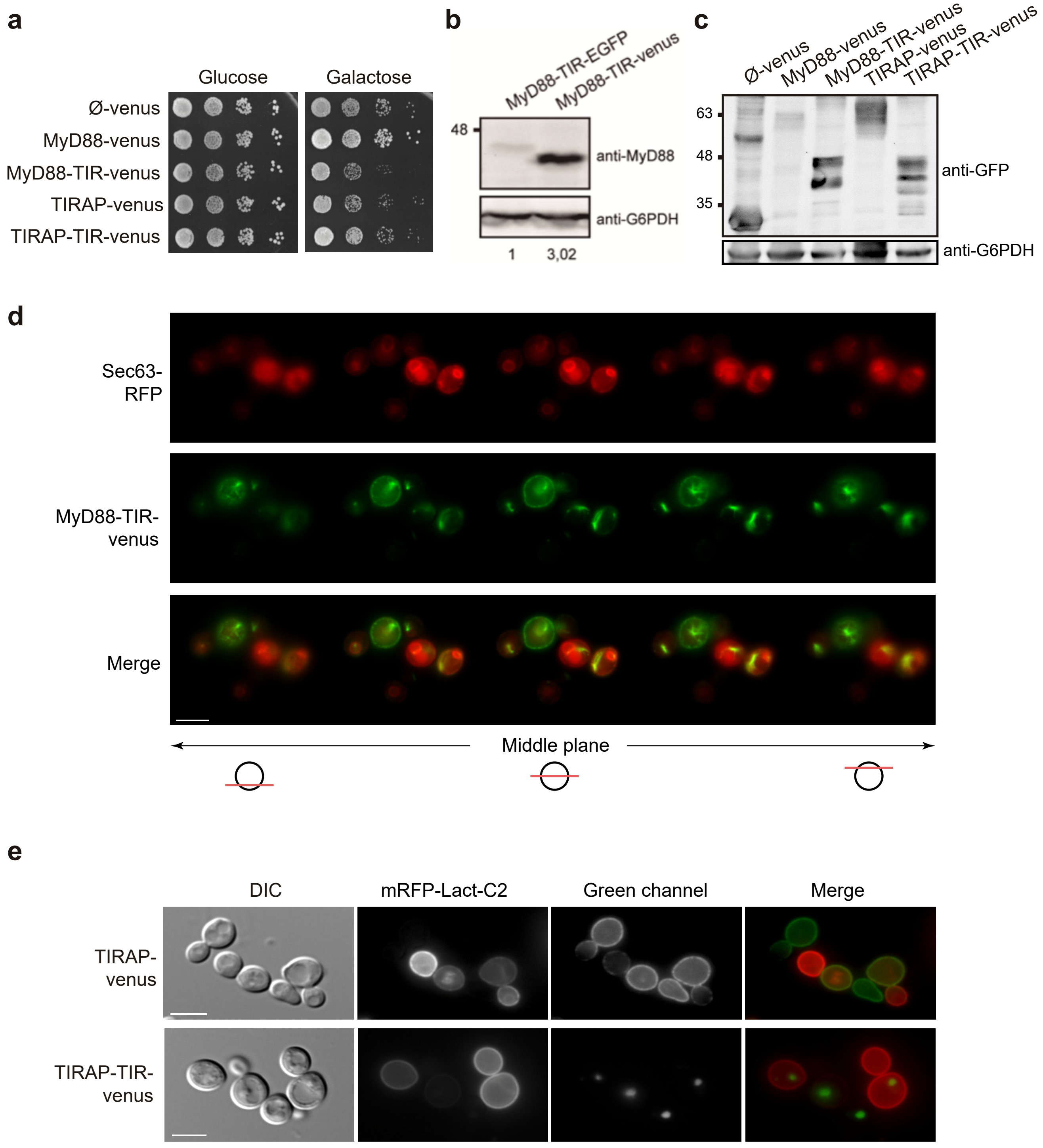

del Val et al. Fig. S8

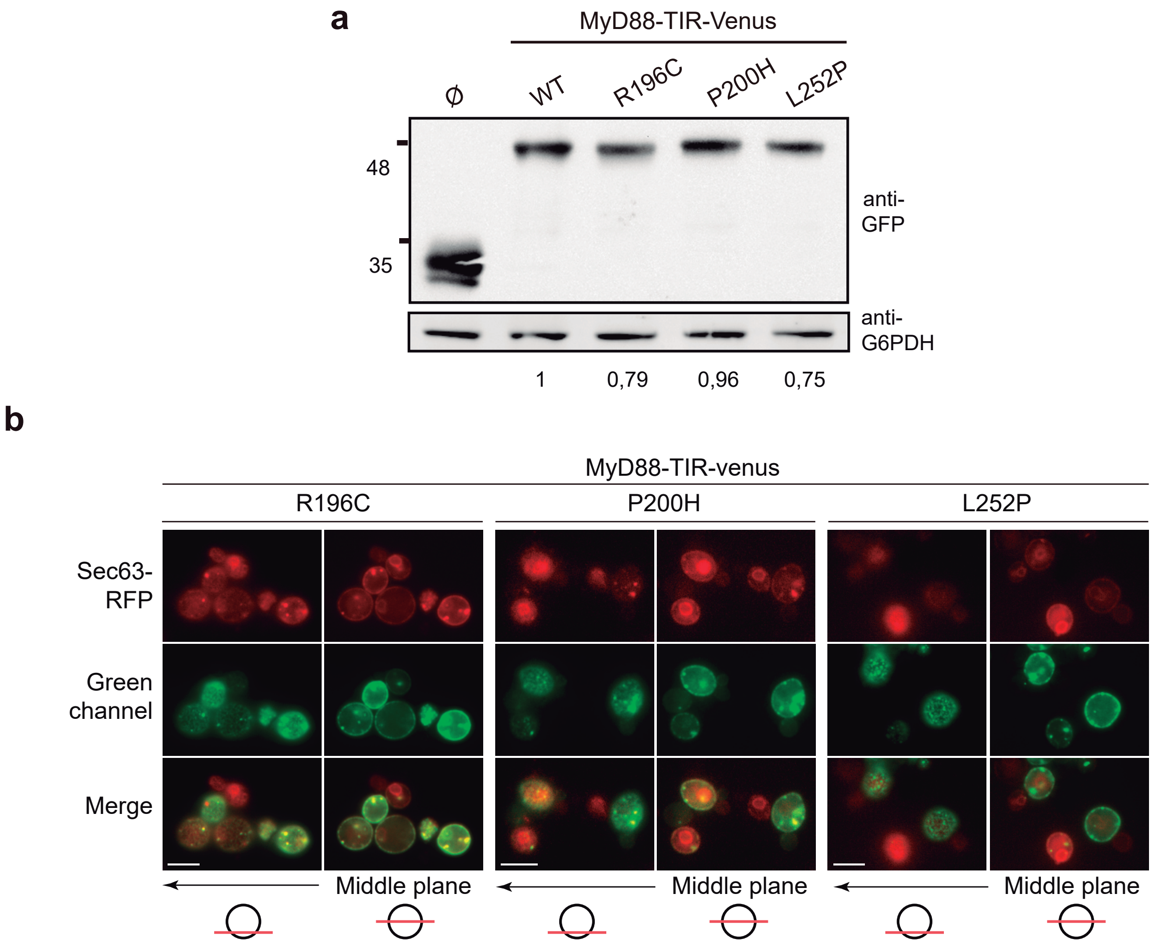

del Val et al. Fig. S9

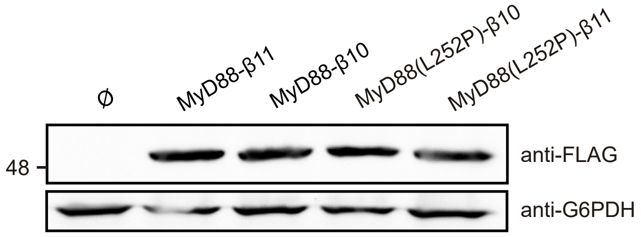

Supplement: Supplementary file 1 — Supplementary Material 1 [file 18_2025_5827_MOESM1_ESM.pdf]
